# A Risk‐Based Framework for Ranking Microbiological Hazards and Identifying Mitigation Strategies for Dried Fruits, Vegetables, Herbs, and Spices Used in Ready‐to‐Eat Foods

**DOI:** 10.1111/1541-4337.70567

**Published:** 2026-08-19

**Authors:** Heidy M. W. den Besten, Linda J. Harris, Jennifer C. Acuff, Francois Bourdichon, Rob Limburn, Andreja Rajkovic, Anett Winkler, Polly D. Courtney

**Affiliations:** ^1^ Food Microbiology Wageningen University & Research Wageningen the Netherlands; ^2^ Department of Food Science and Technology University of California Davis California USA; ^3^ University of Arkansas System Division of Agriculture Fayetteville Arkansas USA; ^4^ SODIAAL International Paris France; ^5^ Università Cattolica Del Sacro Cuore, DiSTAS Piacenza Italy; ^6^ Campden BRI Ltd Chipping Campden UK; ^7^ Laboratory of Food Microbiology and Food Preservation, Department of Food Technology, Safety and Health, Faculty of Bioscience Engineering Ghent University Ghent Belgium; ^8^ Cargill Germany GmbH Düsseldorf Germany; ^9^ General Mills Inc. Minneapolis Minnesota USA

**Keywords:** dried matrices, hazards, ingredients, pathogens, risk‐based ranking

## Abstract

Low water activity ingredients, including dried fruits, vegetables, herbs, and spices, are widely used in ready‐to‐eat (RTE) and non‐RTE foods. Although low water activity limits microbial growth, bacterial pathogens can survive for extended periods in these products. Pathogen virulence, incorporation of dried ingredients in foods, and subsequent handling of the finished foods can collectively contribute to foodborne illness risk. This review summarizes the microbiological hazards associated with dried ingredients and discusses the effectiveness of conventional and emerging decontamination treatments for reducing pathogen levels. To support the evaluation of dried ingredients for RTE and non‐RTE food applications, a qualitative risk‐based microbiological decision framework is presented, along with practical examples demonstrating its use to rank risks and identify appropriate mitigation strategies. The framework considers the ingredient supply chain from production and harvest through packaging of finished products and integrates available outbreak, pathogen prevalence and levels, and product‐specific data. This framework is intended to support the food manufacturing and food service sectors by providing a transparent, structured approach for evaluating, managing, and communicating pathogen risks associated with dry ingredients.

## 1 Introduction

Fruits, vegetables, herbs, and spices are cultivated across a wide range of climates and agricultural systems worldwide (FAO and WHO 2022a, 2022b; ICMSF 2011; Ma et al. 2014; Pinkas and Keller 2014). This geographic diversity is manifested in wide variability in cultivation, harvesting, and postharvest handling practices that range from manual labor to highly automated systems (Ma et al. 2014; Pinkas and Keller 2014; Schaarschmidt et al. 2016). To achieve shelf stability, these commodities are dried using methods that span traditional sun drying to advanced industrial techniques (Bourdoux et al. 2016) that have historically prioritized preservation of sensory attributes and the prevention of microbial or biochemical spoilage. The extended shelf stability afforded by low water activity has facilitated global trade in dried fruits (e.g., apricots, mangos, and raisins), vegetables (e.g., bell peppers, garlic, and onions), herbs (e.g., basil, oregano, and parsley), and spices (e.g., black pepper, cumin, ginger, and turmeric) (Mathot et al. 2021). This has enabled their widespread use as ingredients in ready‐to‐eat (RTE) foods, composite dry products, and a broad range of culinary applications (FAO and WHO 2022a, 2022b).

**FIGURE 1:**
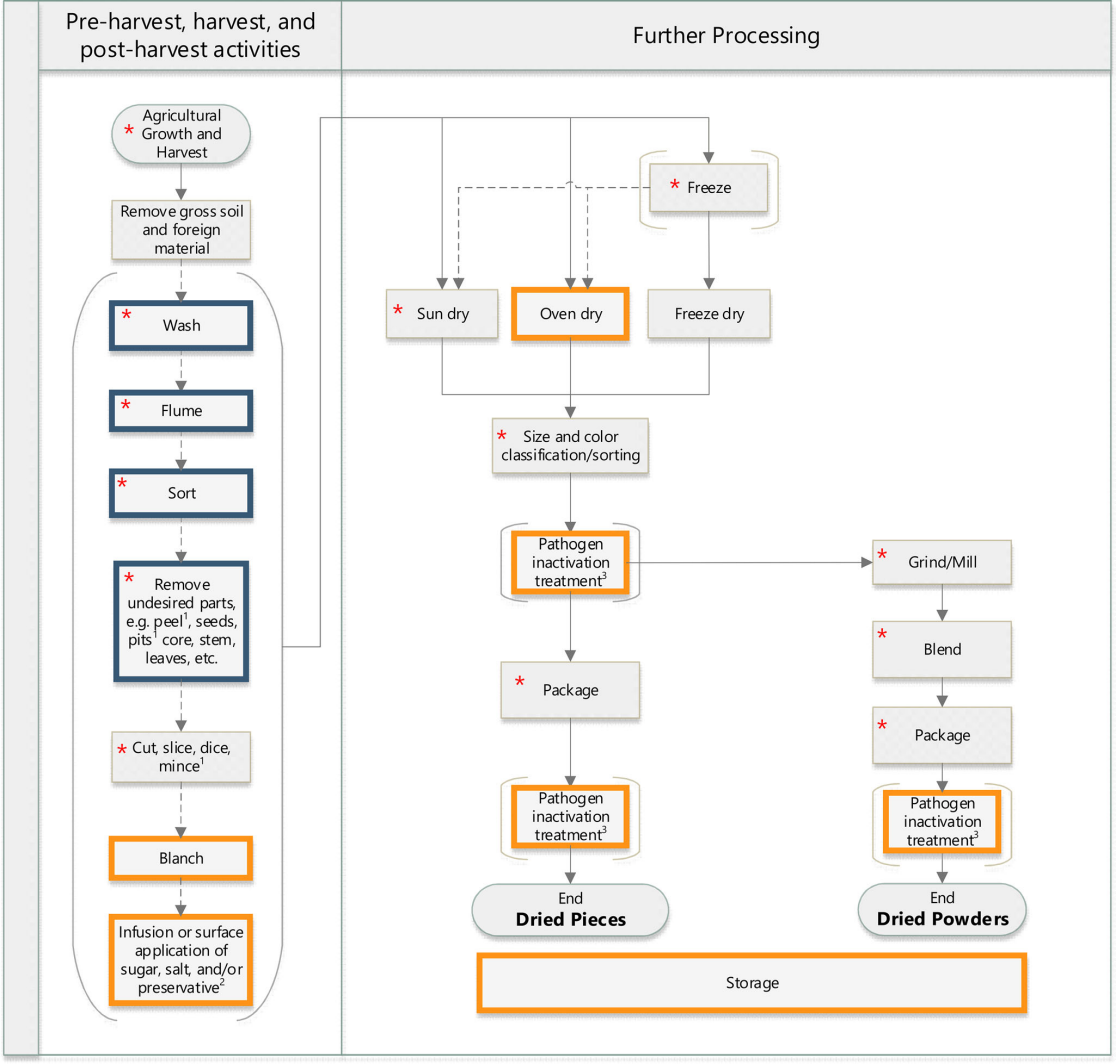
Simplified generic process flow for dried fruits, vegetables, herbs, and spices. The flow diagram is simplified to focus on microbiological contamination and control; therefore, some steps have been intentionally omitted (e.g., receiving, storage, metal detection, and labeling). The order of steps may differ in specific processes. Parentheses around a step and dashed arrows indicate steps that depend on the specific raw material, finished product, and/or manufacturing process. These steps do not occur for all products. Boxes outlined in orange indicate potential pathogen reduction due to lethality effects at those steps. Boxes outlined in blue indicate potential minor pathogen reduction due to physical removal of materials. *Indicates potential sources of pathogen contamination if not managed properly. ^1^Peeling, pit removal, and mincing can be facilitated with steam which would provide additional pathogen reduction beyond just removal of contaminated material. ^2^Preservatives may include sulfur dioxide, sulfites, and ascorbic acid. ^3^Some pathogen inactivation treatments can occur before or after packaging.

Dried ingredients are derived from diverse plant parts, including bark (e.g., cinnamon), flowers (e.g., clove and saffron), fruits (e.g., apricots, chili peppers, and paprika), rhizomes (e.g., ginger), roots (e.g., carrots), and seeds (e.g., cumin and mustard). Following harvest, some products are directly dried, whereas others may be washed, fermented, or subjected to various pretreatments before drying to achieve the shelf stability required for storage, transport, and use in food products (Figure 1, Bourdoux et al. 2016; Mathot et al. 2021).

As natural agricultural products, these ingredients carry microbiota originating from their cultivation environments, including microorganisms associated with soil, plant surfaces, and other environmental sources (Figure 1, FAO and WHO 2022b; Mathot et al. 2021; McKee 1995; Ma et al. 2014; Pinkas and Keller 2014). Production of individual ingredients is often concentrated in a limited number of countries, whereas consumption occurs worldwide, increasing supply‐chain complexity and variability in production, handling, and processing conditions.

Several hygiene and safety challenges persist across production and distribution chains for dry ingredients (Beuchat et al. 2013; FAO and WHO 2022b). Because of their low water activity, dried fruits, vegetables, herbs, and spices have historically been considered to present a low risk for foodborne illness (Gurtler et al. 2014). However, many foodborne pathogens can survive in low water activity foods for extended periods, posing potential risks of illness depending on subsequent handling, dose, and infectivity (Beuchat et al. 2013; CAC 2015; Cuzzi et al. 2021; Chitrakar et al. 2019; FAO and WHO 2022a, 2022b; Gurtler and Keller 2019). Dried fruits, vegetables, herbs, and spices have been linked to foodborne outbreaks since at least 1973 when an outbreak of salmonellosis in Canada was linked to imported black and white pepper (Van Doren et al. 2013). Advances in outbreak investigation, traceability, and detection methods have since revealed a broader range of these products as vehicles for foodborne pathogens (Acuff et al. 2023).

In many food applications, dried ingredients are incorporated at stages of processing that occur after validated pathogen‐reduction steps, such that no additional lethality treatment is applied prior to consumption. This practice is influenced by the widespread use of dried ingredients in minimally processed and “natural” foods as well as RTE products such as cereals and sweet and salty snacks. Consequently, research has focused on evaluating existing technologies and identifying alternative technologies that can reduce pathogen loads without significantly compromising sensory attributes or overall product quality (see, e.g., Bourdoux et al. 2016; Deng et al. 2020). However, these efforts are complicated by the diversity of intrinsic product characteristics, variability in processing technologies, and differences in modeling approaches for microbiological inactivation studies (see, e.g., Den Besten et al. 2018). Options to improve microbiological safety of dried ingredients may exist but are not always available, applied effectively, or legally permitted (see, e.g., Acuff et al. 2020, 2023; Chen et al. 2021; Newkirk et al. 2018; Sádecká 2007; Wei et al. 2019). For example, ethylene oxide treatment, once commonly applied to spices to inactivate microorganisms, is banned in the European Union (EU) and its use in the United States (US) is restricted (US EPA 2025).

The global diversity of applications and regulatory frameworks, coupled with inconsistent definitions of ingredient categories, complicates the assessment of microbiological risks and intervention strategies associated with dried food ingredients. Therefore, this review aims to (1) identify and characterize the microbiological hazards associated with dried fruits, vegetables, herbs, and spices; (2) assess the impact of postharvest handling and drying on hazard mitigation; (3) evaluate factors influencing pathogen survival during storage; (4) review potential intervention strategies; and (5) propose a risk‐based decision framework for suppliers and food business operators. Chemical (including mycotoxins) and physical hazards were considered outside the scope of this review.

## 2 Outbreaks

To date, outbreaks linked to dried herbs and spices have been associated with *Bacillus cereus*, *Clostridium perfringens*, pathogenic *Escherichia coli*, *Salmonella enterica*, and norovirus (Table 1; Gurtler et al. 2019; Van Doren et al. 2013). In contrast, outbreaks associated with dried fruits and vegetables have primarily involved *Salmonella* (Table 1; dried mixed fruit, dried vegetable spice or seasoning mix, and dried mushrooms) and foodborne viruses (hepatitis A and norovirus in dried fruit, dried tomatoes, and dates). The outbreaks often span several months, and root causes of contamination are seldom identified. Many outbreaks involve imported products, sometimes in mixtures of ingredients from more than one country. Table 1 summarizes the pathogens implicated in outbreaks and the associated dried fruits, vegetables, herbs, and spices between 2000 and 2023, though complete outbreak reports are not always available. Information on salmonellosis outbreaks is generally more complete than for other pathogens, and some root‐cause investigation reports highlight critical control information, or pathogen and/or food characteristics that influenced disease outcomes, which are further described in this section.

**TABLE 1:** Foodborne disease outbreaks associated with naturally contaminated dried fruits, vegetables, herbs, and spices between 2000 and 2023.

Pathogen and food commodity	Year	Country	Cases (total, hospitalized, deaths)	Remarks	Reference ^a^
*Bacillus cereus*					
Unspecified spices (taco)	2000	US	4, 1, 0	Confirmed etiology	NORS database
Unspecified spices (blue cheese dressing)	2000	US	2 cases	Suspected etiology	NORS database
Unspecified herbs and spices	2007	France	146 cases		EFSA (2013)
Curry	2009	Belgium	7 cases		EFSA (2013)
White pepper	2010	Denmark	112 cases		EFSA (2013)
Turmeric (curcuma)	2011	Finland	2 outbreaks (19 and 4 cases)		EFSA (2013)
Cumin, ground	2011	Finland	3 cases		EFSA (2013)
Pepper	2011	Denmark	52 cases		EFSA (2013)
*Clostridium perfringens*					
Unspecified herbs and spices	2007	France	19 cases		EFSA (2013)
Shiga toxin‐producing *Escherichia coli* (STEC)					
Unspecified herbs and spices	2012	EU	Unreported	Strong‐evidence outbreak ^b^	EFSA and ECDC (2014)
Non‐STEC pathogenic *E. coli*					
Unspecified herbs and spices	2013	EU	Unreported	Strong‐evidence outbreak	EFSA and ECDC (2015a)
Hepatitis A					
Dates, genotype IIIA	2018, 2019	Denmark, Sweden	9, 27, and 1 case in Sweden, Denmark, Norway, respectively	Dates from Iran suspected. In 2018, one of the outbreak strains was detected in dates.	Whitworth (2019b)
Dates, genotype IB	2021	Australia	6, 4, 0	Dates from Jordan; genomically identical to 2021 UK outbreak strain). HAV genotype IB detected in 2/10 25‐g retail subsamples. Batches linked to Australia and England/Wales outbreaks were harvested months apart from different Jordan River farms.	O'Neill et al. (2022), Whitworth (2022)
Dates, genotype IB	2021	England, Wales	31, 25, 0	Dates from Jordan. HAV genotype IB was detected in two samples of dates.	Garcia Vilaplana et al. (2021)
Dried fruit (suspect), genotype IB	2005	Turkey	4 cases	Danish cases had travelled to Turkey and stayed in same resort.	Howitz et al. (2005)
Tomatoes (semi‐dried also known as “sun dried”), genotype IB	2009	Australia	562, ∼253, 1	HAV genotype IB RNA detected in 22 samples. Outbreak strains in the Netherlands and Australia were identical. Imported products implicated, supported by similar outbreaks in France and the Netherlands.	Donnan et al. (2012)
Tomatoes (semi‐dried also known as “sun dried”), genotype IB	2011	UK	4 cases suspect or epidemiologically linked	Possibly linked to 2011 the Netherlands outbreak; 2 cases plus 2 cases with different sequences.	Carvalho et al. (2012)
Tomatoes (semi‐dried also known as “sun dried”), genotype IB	2011	The Netherlands	7 confirmed and 1 probable case (includes secondary suspect cases)	HAV genotype IB strain identical to strains associated with earlier outbreaks in Australia and Europe; case isolates closely related to strains in travellers returning from the Middle East.	Fournet et al. (2012)
Tomatoes (semi‐dried, in oil), genotype IB	2009–2010	The Netherlands	13 cases (+ 4 related secondary cases)	HAV genotype IB strain was closely related to a strain from a 2009 Australian outbreak.	Petrignani et al. (2010)
Tomatoes (semi‐dried, in oil), genotype IB	2009–2010	France	59, 28, 0; 12 cases were probably secondary cases	Semi‐dried tomatoes (received frozen, and then mixed with oil and herbs) imported from Turkey were implicated.	Couturier et al. (2011), Gallot et al. (2011)
Norovirus					
Dried fruit (suspect)	2002	Spain	48, 5, 0	Strawberry jam, butter, dried fruit were associated. Hypothesis: foods were contaminated by handlers who reported being sick before the outbreak started.	Eriksen et al. (2004)
Unspecified herbs and spices	2019	Italy	13 cases	Strong‐evidence outbreak	EFSA and ECDC (2021)
*Salmonella enterica* subsp. *enterica*					
Dried fruit and coconut snack mix, serovar Agbeni	2018–2019	Norway	56, 21, 0	WGS confirmed outbreak strain isolated from consumed mix; Fruits sourced from Ghana, Philippines, Thailand, Turkey; packed in Italy, distributed to 6 European countries (including Norway); no other countries reported cases.	Johansen et al. (2021), Whitworth (2019a)
Aniseed (herbal tea), serovar Agona	2002	Germany	39, 21, 0	Nationwide infant outbreak with multiple tea brands traced to one importer. Aniseed batch fertilized with manure. Tea usually prepared with boiling water; some cooled rapidly before consumption. Estimated levels: 0.036 MPN/g.	Koch et al. (2005)
Curry powder, serovar Braenderup	2002	United Kingdom	20, 1, 0	Serovar Braenderup was detected in curry powder added to an egg dish that was kept at room temperature before serving.	Van Doren et al. (2013)
Unspecified herbs and spices	2006	EU	16, 2, 0		EFSA and ECDC (2007)
Dried vegetable spice mixes, serovar Enteritidis	2014–2015	Sweden	174 cases	Spice mix containing dried vegetables tested positive for the outbreak strain.	Jernberg et al. (2015)
Unspecified herbs and spices	2012	EU	Unreported	Strong‐evidence outbreak	EFSA and ECDC (2014)
Unspecified herbs and spices	2013	EU	Unreported	Strong‐evidence outbreak	EFSA and ECDC (2015a)
Red and black pepper (in ready‐to‐eat salami), serovars Montevideo and Senfenberg	2009	US (multistate)	283, 52, 0. Montevideo (272), Senftenberg (11)	Pepper added after CCP step. Median age 37 years (<1–93 years). Outbreak strain (Montevideo) found in floor drain. Serovars isolated from intact pepper containers: outbreak Montevideo and Senftenberg; Bareily, Newport, and Virchow.	Gieraltowski et al. (2013), Van Doren et al. (2013), NORS database
White pepper, ground, serovar Rissen	2008	US (multistate)	87, 8, 1	3 populations: Cases linked to Asian restaurants (white pepper in table shakers); cluster at resort buffet; hospital‐associated cases exposed to cold salads and some hot foods, older cases (median age 74 years) with comorbidities.	Higa (2011), NORS database
Fennel seed (tea), serovar Senftenberg	2007	Serbia	14, 4, 0	Seeds from individual producers. Cases: 10 infants <12 month, (2 hospitalized) and 4 adults (3 mild infection; 1 with concomitant *Clostridium difficile* infection, hospitalized). Parents of 3 infants: tea prepared with boiling water.	Ilic et al. (2010)
Dried wood ear mushrooms, serovar Stanley	2020	US (multistate)	55, 6, 0	Imported mushrooms sold to restaurants. Mushrooms are soaked before using or included in noodles.	CDC (2020), US FDA (2020)
Seasoning mix with broccoli and parsley powders, multiple serovars	2007	US (multistate)	18, 2, 0 (Typhimurium); 69, 6, 0 (Wandsworth)	Broccoli and parsley powders in puffed rice and corn snack food seasoning. Serovars isolated from: finished product (Typhimurium, Wandsworth, Haifa); seasoning mix (Wandsworth); broccoli powder (Wandsworth); parsley powder (Mbandaka)	CDC (2007), Sotir et al. (2009)
Moringa leaf powder, serovar Virchow	2015	US (multistate)	35, 6, 0	Raw ingredient imported from South Africa. No reduction step applied. Product marketed as raw organic meal replacement shake not intended to be cooked prior to consumption.	Gambino‐Shirley et al. (2018), NORS database
Unspecified herbs and spices	2015	EU	184 cases	Strong‐evidence outbreak	EFSA and ECDC (2016)
Unspecified herbs and spices	2017	EU	Unreported	Strong‐evidence outbreak	EFSA and ECDC (2018)

Abbreviations: CCP, critical control point, EU, European Union; HAV, Hepatitis A virus; UK, United Kingdom; US, United States; WGS, whole genome sequencing.

^a^ NORS, National Outbreak Reporting System (US), Bacteria, Enterics, Ameba, and Mycotics (BEAM) dashboard; https://www.cdc.gov/ncezid/dfwed/BEAM‐dashboard.html

^b^ “Strong‐evidence” outbreaks are described as those where more detailed information is collected, including food vehicle and its origin, nature of evidence linking the outbreak cases to the food vehicle, type of outbreak, setting, place of origin of the problem and contributory factors.

In 2002 and 2007, herbal teas were implicated in outbreaks of salmonellosis in Germany and Serbia, respectively (Ilic et al. 2010; Koch et al. 2005). In both outbreaks, reported tea‐brewing practices of the cases involved rapid cooling of the boiling water used for preparation. Hospitalized cases included infants and an adult with a concomitant infection. Matching *Salmonella* strains were recovered from patients, herbal tea samples, and unprocessed fennel seeds (Ilic et al. 2010) or aniseed (Koch et al. 2005) confirming seeds as the contamination source. These findings demonstrate that contaminated dried ingredients can serve as the source of contamination of the final product when no effective pathogen‐reduction step is applied prior to consumption.

Curry powder and ground white pepper were implicated in *Salmonella* outbreaks in 2002 (the United Kingdom; Van Doren et al. 2013) and 2008 (multistate outbreak in the US; Higa 2011) where the spices were used as ingredients in cold dishes. *Salmonella* was implicated in a multistate outbreak in the US in 2015, where *Salmonella* Virchow was isolated from raw moringa leaf powder that was used as an ingredient in shelf‐stable blended powdered product intended to be rehydrated and consumed as a drinkable meal replacement (Gambino‐Shirley et al. 2018). No pathogen reduction step was applied to the ingredients or the finished product that was not intended to be cooked before consumption. Red and black pepper were implicated in a multistate outbreak in the US in 2009, with fermented salami as the food vehicle; pepper was added after the critical control point for pathogen reduction (Gieraltowski et al. 2013; Van Doren et al. 2013). *Salmonella* serotypes Montevideo and Senftenberg were associated with the reported cases, although other serotypes (e.g., *Salmonella* Bareilly, Newport, and Virchow) were also isolated from pepper samples. These outbreaks highlight the risk of adding dried ingredients to RTE foods after a critical process control step.

An outbreak of *Salmonella* Agbeni was identified in Norway in 2019, with a dried fruit mix as the contaminated commodity (Johansen et al. 2021; Whitworth 2019a). One of the 56 cases had a dual infection with *Salmonella* Agbeni and *Salmonella* Wagenia. The dried fruit mix, contained fruits from different countries and continents, was packaged in Italy, and distributed to several European countries, although cases were only reported in Norway. Nine opened samples of mixed fruits were collected from case homes, and two unopened packages were collected from grocery stores. The outbreak strain *Salmonella* Agbeni (confirmed by whole genome sequencing [WGS]) was isolated from all opened and one unopened package (sample size not indicated) leading to a product recall. *Salmonella* Gamaba was isolated from both unopened packages. Finished product samples tested at the Italian firm were negative for *Salmonella*, as were supplier and health authority tests of the banana, coconut, papaya, and pineapple ingredients, highlighting the limitations of finished‐product testing for preventing foodborne illness, particularly in dried products with mixed ingredients.

A multistate outbreak of salmonellosis in the US in 2020 was linked to consumption of imported dried wood ear mushrooms (also known as black fungus or jelly ear; *Auricularia auricula‐judae*) that had been distributed to restaurants but not sold at retail (CDC 2020; US FDA 2020). There were 55 illnesses and six hospitalizations in six states over 8 months. Mushroom samples from one of the restaurants where cases had eaten tested positive for *Salmonella*, and WGS confirmed the isolates as the outbreak strain (*Salmonella* Stanley). Dried mushrooms can be incorporated into a variety of savory dishes and are typically soaked in liquid (e.g., water, broth) before use. After the outbreak, FDA recommended rehydrating mushrooms using boiling water (US FDA 2020). A subsequent study of 25 restaurant chefs or owners who used wood ear mushrooms (Chen et al. 2024) found that the mushrooms were commonly rehydrated with cold water.

Dried vegetables (Jernberg et al. 2015) and a vegetable seasoning mix (Sotir et al. 2009; CDC 2007) were associated with outbreaks of *Salmonella* Enteritidis phage type 13a in Sweden and *Salmonella* Wandsworth and *Salmonella* Typhimurium in the US, respectively. The *Salmonella* Enteritidis outbreak spanned 7 months during 2014–2015, and most cases (108/174) were linked to a single restaurant. *Salmonella* Enteritidis phage type 13a infections are rare in Sweden (<1%). *Salmonella* was detected in an unopened package of spice mix (product contained dried onions, carrots, and parsnips) collected from the restaurant as well as from a pastry bag used for piping mashed potatoes that contained the spice mix. The isolates from the spice mix (three) and the pastry bag (one) had the same Multiple‐Locus Variable‐Number Tandem Repeat Analysis (MLVA) profile as 43 of the cases. Additional testing of opened packages of the spice mix also yielded *Salmonella* that matched the outbreak strain by MLVA. It was estimated that there were very low concentrations of the pathogen in the mix, underscoring that low levels of contamination can result in illness and that cases may occur over extended periods, particularly in products with long shelf lives, such as dried vegetables and dry mixes.

Cases of *Salmonella* Wandsworth (*n* = 69) and *Salmonella* Typhimurium (*n* = 18) from 23 US states were linked to consumption of an RTE puffed rice and corn snack coated with dried vegetables (Veggie Booty) over a period of more than 4 months in 2007. Most cases (93%) occurred in children under 3 years of age. A total of 26 bags of the implicated product were collected from case–patient households and 35 bags from retail stores. *Salmonella* Wandsworth was recovered from two opened and nine unopened bags with a wide range of best‐by dates. *Cronobacter sakazakii*, *Salmonella* Kentucky, and *Salmonella* Typhimurium were also isolated from unopened bags, whereas *Salmonella* Haifa, *Salmonella* Typhimurium, and *Salmonella* Wandsworth were recovered from finished product samples at the production facility. These findings demonstrate that multiple pathogens may be associated with a single contaminated product. The product was prepared by blending grains and other ingredients into a paste that was extruded (a thermal kill step) to form the puffed product. The seasoning mix was applied to the surface after extrusion, with no further kill step, highlighting the need for evaluating the risks associated with addition of seasoning mixes or other ingredients to RTE foods.

Foodborne viruses do not replicate in foods, but ingredients that are directly handled by humans during processing are susceptible to contamination. A norovirus outbreak linked to unspecified spices and herbs was reported in 2019 (EFSA and ECDC 2021). Hepatitis A virus (HAV) has been associated with two outbreaks involving dates (Garcia Vilaplana et al. 2021; O'Neill et al. 2022; Whitworth 2019b, 2022) and, separately, with dried tomatoes (Carvalho et al. 2012; Couturier et al. 2011; Donnan et al. 2012; Fournet et al. 2012; Gallot et al. 2011; Petrignani et al. 2010) where outbreak strains isolated from Australia and European countries were identical or closely related. The first reported date‐associated outbreak involved HAV genotype IIIA cases in Denmark and Sweden, a genotype typically linked to travel‐associated cases in Scandinavia. In Denmark, 27 cases were reported in 2018; four different strains of genotype IIIA were detected in eight confirmed cases, and the virus was detected in a single date sample. In 2019, Sweden (nine cases) and Norway (one case) reported infections with strains of genotype IIIA in Swedish cases that were similar to those from the 2018 Danish outbreak. Dates imported from Iran were suspected in the Swedish outbreak, although the virus was not detected in the product. These outbreaks demonstrate how global distribution of dried ingredients can result in foodborne illnesses occurring across widely dispersed geographic regions.

## 3 Prevalence and Levels of Pathogens

Different data sources were used to evaluate prevalence and levels of pathogens in dried fruits, vegetables, herbs, and spices. Data were extracted from published literature and surveys, as well as from European Food Safety Authority (EFSA) reports and from the EU Rapid Alert System for Food and Feed (RASFF) information portal between 2020 and 2024. The EU RASFF portal was established to allow food safety authorities within the EU to rapidly exchange information on health risks derived from food or feed. Data on microbial contamination come from imported and domestic product checks, food facility testing, and foodborne outbreak investigations (Banach et al. 2016). Although access to RASFF is exclusively for member authorities, a publicly available summary is available in the so‐called RASFF window. That database can be searched for notifications made in 2020 and later.

RASFF revealed very few notifications for dried fruits and vegetables compared to that of herbs and spices. Beyond RASFF information, pathogen prevalence data for dried fruits and vegetables largely originate from retail/market surveys (Table 2), except for a single study where samples were taken at a food processor (Witthuhn et al. 2005). In several of the retail surveys, sample numbers and individual sample sizes are low (e.g., less than 20 samples, <25 g), making it difficult to calculate prevalence rates with statistical relevance. In contrast, most pathogen prevalence data for herbs and spices were found in EFSA reports or a US Food and Drug Administration (US FDA) risk profile for pathogens in spices (US FDA 2017). These data include information on prevalence and levels of *B. cereus, Cronobacter* spp., pathogenic *E. coli*, *Listeria monocytogenes*, and *Salmonella*, with the latter microorganisms most often reported.

**TABLE 2:** Prevalence of pathogens on naturally contaminated dried fruits, vegetables, herbs, and spices between 2003 and 2023.

**Pathogen and** **sample collection**	**Year**	**Product**	**Sample size**	**Positive samples/total samples; levels provided when determined or available**	**Reference; comments**
*Bacillus cereus*					
Wales; retail, food service	2003–2005	Dried fruits	NA	0/555, 4‐5 log CFU/g; 2/555, <4 log CFU/g	Meldrum et al. (2006)
New Zealand; retail	2004	Dried potato flakes	NA	13/50, <10 CFU/g; 8/50, >100 CFU/g; 1/50, 2.5 log CFU/g (highest count)	Turner et al. (2006)
Maseru, Lesotho; retail and informal retail (home prepared)	2014	Commercial dried fruits	NA	0/164, <10 CFU/g	Ntuli et al. (2017)
Maseru, Lesotho; retail and informal retail (home prepared)	2014	Home dried fruits (apples, peaches)	NA	Presumptive mean levels 1.7 log CFU/g (20 apples), 1.3 log CFU/g (20 peeled peaches), <10 CFU/g (20 unpeeled peaches)	Ntuli et al. (2017)
Maseru, Lesotho; retail and informal retail (home prepared)	2014	Home dried vegetables (pumpkin leaves, spinach, *Wahlenbergia androsacea*)	NA	Presumptive mean levels 1.8 log CFU/g (15 pumpkin leaves), 1.8 log CFU/g (15 spinach), 1.7 log CFU/g (15 *W. androsacea*)	Ntuli et al. (2017)
Korea; retail	Unspecified	Dried red pepper, red pepper powder	NA	15/28 (dried red pepper); 10/12 (red pepper powder)	Park et al. (2022)
Bavaria; retail assumed	2007–2013	Dried mushrooms	10 g	110/135 presumptive *B. cereus*; 11/135 emetic type	Messelhäusser et al. (2014)
Switzerland; unspecified	Unspecified	Powdered mashed potato products	10 g	12/13	Heini et al. (2018)
*Bacillus cytotoxicus*					
Switzerland; retail	2018, 2020	Mashed potato powder	10 g	19/20	Burtscher et al. (2021)
Germany; retail assumed	Unspecified	Dried potato products	10 g	15/17 (mashed potato power/ flakes/ granules); 8/12 (other potato powders/ flakes/ granules)	Contzen et al. (2014)
Belgium or West Africa; retail	Unspecified	Range of dried mushroom, onion and potato products	10 g	20/20 (potato flakes); 1/6 (potato peelings); 0/1 (mushroom, potato flour, potato slices, onion powder); 0/2 (dried onion)	Koné et al. (2019)
*Bacillus* spp.					
USA; online (product from China with samples from Japan, Thailand, and US)	2013–2015	Dried shiitake mushroom (whole, sliced, powdered)	NA	Mean levels 1.8 ± 0.5 log CFU/g (20 whole), 1.5 ± 0.3 log CFU/g (28 sliced), 1.4 ± 0.7 log CFU/g (14 powdered)	Kim et al. (2016)
*Clostridium botulinum* (proteolytic and non‐proteolytic)					
China – 21 cities; retail	2008	Dried mushrooms	10–25 g	0/27 (shiitake); 0/21 (wood ear)	Malakar et al. (2013)
*Cronobacter*					
Prague, CZ; retail	2010	Dried herbs, spices, vegetables	25 g	4/10 (basil); 2/15 (caraway); 0/11 (cinnamon); 3/10 (ginger); 2/12 (marjoram); 0/10 (oregano); 1/14 (pepper); 0/12 (pepper, sweet); 2/10 (pimento); 0/14 (potato dumpling powder)	Hochel et al. (2012)
Warsaw, Poland; retail	Unspecified	Basil, tarragon, parsley	10 g	9/38	Garbowska et al. (2015)
Warsaw, Poland; retail	2018–2019	Candied and dried fruits	25 g	0/8 (candied); 0/24 (dried)	Berthold‐Pluta et al. (2021)
Shiga toxin–producing *Escherichia.coli*, including O157:H7					
EU; retail; processor	2011	Herbs and spices	Various; 25 g	0/88; 0/31	EFSA and ECDC (2013)
EU; unspecified	2018	Herbs and spices	NS	3/429	EFSA and ECDC (2019)
EU; unspecified	2019	Herbs and spices	NS	2/685	EFSA and ECDC (2021)
Hepatitis A					
The Netherlands; retail	2009–2010	Figs	30 g	0/91 PCR only	Boxman et al. (2012)
The Netherlands; retail	2009–2010	Dates	30 g	2/185 PCR only, genotype 1A	Boxman et al. (2012)
*Listeria monocytogenes*					
Wales; retail, food service	2003–2005	Dried fruits (undefined)	NA	0/555 >100 CFU/g	Meldrum et al. (2006)
EU; retail	2012	Herbs and spices	25 g; NA	0/67; 2/105 <100 CFU/g ^a^, 0/105 >100 CFU/g	EFSA and ECDC (2014)
EU; processor	2012	Herbs and spices	NA	0/46 <100 CFU/g ^a^, 0/46 >100 CFU/g	EFSA and ECDC (2014)
EU; retail	2013	Herbs and spices	Various; NA	0/43; 0/51 <100 CFU/g ^a^, 0/51 >100 CFU/g	EFSA and ECDC (2015a)
EU; processor	2013	Herbs and spices	Various; NA	2/72; 2/45 <100 CFU/g ^a^, 0/45 >100 CFU/g	EFSA and ECDC (2015a)
EU; unspecified	2013	Herbs and spices	25 g; NA	0/75; 0/70 <100 CFU/g ^a^, 0/70 >100 CFU/g	EFSA and ECDC (2015a)
EU; retail	2014	Herbs and spices	NS; NA	0/48; 0/109 <100 CFU/g ^a^, 0/109 >100 CFU/g	EFSA and ECDC (2015b)
EU; processor	2014	Herbs and spices	NS; NA	17/282; 0/33 <100 CFU/g ^a^, 0/33 >100 CFU/g	EFSA and ECDC (2015b)
EU; unspecified	2014	Herbs and spices	NA	0/10 < 100 CFU/g ^a^, 0/10 > 100 CFU/g	EFSA and ECDC (2015b)
EU; retail	2015	Herbs and spices	NS; NA	0/19; 0/155 <100 CFU/g ^a^, 0/155 >100 CFU/g	EFSA and ECDC (2016)
EU; processor	2015	Herbs and spices	NS; NA	0/68; 0/23 <100 CFU/g ^a^, 0/23 >100 CFU/g	EFSA and ECDC (2016)
EU; unspecified	2015	Herbs and spices	NA	0/73 <100 CFU/g ^a^, 0/73 >100 CFU/g	EFSA and ECDC (2016)
EU; unspecified	2016	Herbs and spices	NS	0/48	EFSA and ECDC (2017)
EU; unspecified	2017	Herbs and spices	NS	0/45	EFSA and ECDC (2018)
EU; unspecified	2018	Herbs and spices	NS	0/121	EFSA and ECDC (2019)
EU; unspecified	2019	Herbs and spices	NS	2/291	EFSA and ECDC (2021)
*Salmonella*					
Worcester, South Africa; food processor	Unspecified	Dried fruits	NA	0/3 (apricots); 0/4 (nectarines/peaches); 1/3 (prunes); 1.6 log CFU/g; 1/3 (raisins); 1.0 log CFU/g	Witthuhn et al. (2005); Directly plated onto Brilliant Green Agar (BGA)
Wales; retail, food service	2003–2005	Dried fruits (undefined)	25 g	0/555	Meldrum et al. (2006)
EU; unspecified	2004	Herbs and spices	NS	0.9%‐7% (unspecified numbers of samples)	EFSA and ECDC (2005)
EU; unspecified	2005	Herbs and spices	NS	4/129‐4/55	EFSA and ECDC (2006)
EU; unspecified	2006	Herbs and spices	25 g	5/154	EFSA and ECDC (2007)
EU; unspecified	2007	Herbs and spices	25 g	6/1,377	EFSA and ECDC (2009)
Lucknow, India; retail markets	2007	Raisins	NA	4/10 (open samples), (1.0‐2.7 log CFU/g); 2/10 packaged samples, (1.0 and 4.7 log CFU/g)	Sharma et al. (2008); Directly plated onto BGA
EU; unspecified	2008	Herbs and spices	25 g	74/3,403	EFSA and ECDC (2010)
EU; unspecified	2009	Herbs and spices	25 g	63/1,857	EFSA and ECDC (2011)
EU; retail	2010	Herbs and spices	Various	43/2,666	EFSA and ECDC (2012)
EU; processor	2010	Herbs and spices	Various	0/36	EFSA and ECDC (2012)
EU; retail; processor; unspecified	2011	Herbs and spices	Various; 25 g; 25 g	7/1,128; 2/187; 1/127	EFSA and ECDC (2013)
EU; retail; processor; unspecified	2012	Herbs and spices	Various; 25 g; 25 g	5/1,240; 1/262; 36/618	EFSA and ECDC (2014)
US; at entry	2012	Herbs and spices	750 g	1/20 (basil); 15/223 (whole and ground black pepper); 17/92 (coriander, ground); 11/130 (cumin); 7/177 (curry powder); 1/59 (garlic powder); 8/78 (oregano); 3/85 (paprika powder); 36/337 (red pepper powder); 3/50 (white pepper powder)	US FDA (2017); Enumeration data only available for capsicum in 2010 (0.06‐9 MPN/100 g and ground)
EU; retail; processor; unspecified	2013	Herbs and spices	Various; 25 g; 25 g	4/1,310; 5/433; 6/2,552	EFSA and ECDC (2015a)
US; retail	2013–2015	Herbs and spices	125 g	1/529 (basil); 3/1264 (black peppercorn); 3/543 (coriander, ground); 0/549 (cumin); 1/518 (curry powder); 3/615 (garlic powder); 1/669 (oregano); 2/816 (paprika, ground); 4/633 (red pepper, ground); 0/588 (white pepper, ground)	Zhang et al. (2017)
EU; retail; processor; unspecified	2014	Herbs and spices	Various; 25 g; 25 g	2/1,228; 4/347; 3/4,363	EFSA and ECDC (2015b)
Maseru, Lesotho; retail	2014	Commercially dried fruits	NA	0/164 (LOD = 10 CFU/g)	Ntuli et al. (2017); Directly plated onto BGA (LOD = 10 CFU/g)
Maseru, Lesotho; informal retail (home prepared)	2014	Home dried fruits	NA	0/20 (apple); 0/40 (peach)	Ntuli et al. (2017); Directly plated onto BGA
Maseru, Lesotho; retail and informal retail (home prepared)	2014	Home dried vegetables	NA	0/15 (spinach); 15 (*Wahlenbergia androsacea*, 1.0 log CFU/g); 15 (pumpkin leaves, 1.5 log CFU/g)	Ntuli et al. (2017); Directly plated onto BGA
EU; retail; processor; unspecified	2015	Herbs and spices	Various; 25 g; 25 g	10/1,166; 0/226; 8/218	EFSA and ECDC (2016)
Querétaro, Mexico; markets	2015	Raisins and tomatoes	25 g	20/70 (raisins); 37/70 (sun dried tomatoes)	Aguilar‐Vázquez et al. (2022)
EU; unspecified	2016	Herbs and spices	NS	21/1,390	EFSA and ECDC (2017)
EU; unspecified	2017	Herbs and spices	NS	11/2,631	EFSA and ECDC (2018)
EU; unspecified	2018	Herbs and spices	NS	22/2,440	EFSA and ECDC (2019)
EU; unspecified	2019	Herbs and spices	NS	7/2,136	EFSA and ECDC (2021)
*Staphylococcus aureus*					
Wales; retail, food service	2003–2005	Dried fruits (undefined)	25 g	0/555 ≥4 log CFU/g	Meldrum et al. (2006); Plated directly onto Baird‐Parker Agar (BPA), LOD 20 CFU/g
Lucknow, India; market	2007	Raisins	NA	0/10 (open samples); 0/10 (packaged samples)	Sharma et al. (2008); Plated directly onto BPA, LOD = 100 CFU/g
Worcester, South Africa; food processor	unspecified	Dried fruits (high moisture)	NA	≥10 CFU/g: 0/3 (apricots); 3/3 (raisins); 0/3 (prunes); 0/4 (nectarines, peaches)	Witthuhn et al. (2005); Plated directly onto BPA (LOD = 10 CFU/g)

Abbreviations: EU, European Union; LOD, level of detection; NA, not applicable (enumeration rather than enrichment); NS, not specified; US, United States

^a^ Positive at either <100 CFU/g or >100 CFU/g.

However, some of these data may be biased due to investigations driven by reported foodborne outbreaks such as the targeted analysis of fenugreek seeds for STEC in the aftermath of a deadly 2011 *E. coli* outbreak in Germany (sprouted fenugreek seeds; BfR 2011). Therefore, low numbers of notifications, recalls, and prevalence data could also be an artifact of limited investigation.

For this reason, the prevalence of pathogens in many dried commodities is largely unknown because systematic or standardized studies (those with standardized sample weights and sample sizes, comparing a single product from different regions or years of production) have not been published. However, the currently available data allow for a preliminary ranking among the products and for the pathogens analyzed. In that context, the Food and Agriculture Organization of the United Nations (FAO) Microbiological Risk Assessment No. 27 (FAO 2022b) for aromatic herbs and various spices states that “*Salmonella* spp. and the spore‐forming organisms *B. cereus* and *C. perfringens* should be considered foodborne pathogens of particular concern with respect to spices and dried aromatic herbs.” That publication also provides a risk ranking approach for the spices and aromatic herbs included in the study.

### 3.1 *B. cereus* and Other Spore‐Former Prevalence, Levels, and Recall Data

The presence of *B. cereus* (Heini et al. 2018; Messelhäusser et al. 2014) or *Bacillus cytotoxicus* was measured using an enrichment of 10 g samples in several studies (Burtscher et al. 2021; Contzen et al. 2014; Koné et al. 2019) (Table 2). More than 70% of dried mushrooms and powdered mashed potato products were positive for these organisms. In other surveys, *B. cereus* was directly enumerated and counts of *B. cereus* in a variety of commercially dried fruits ranged from undetected (<10 CFU/g) to 1.3–1.7 log CFU/g in home‐dried apples and peaches (Ntuli et al. 2017), <1.0–2.5 log CFU/g in dried potato flakes (Turner et al. 2006), and 1.8 log CFU/g in home dried leafy vegetables (Ntuli et al. 2017). Two RASFF notifications were attributed to *B. cereus*, both of which were related to dried mushrooms. Other spore‐forming bacteria have also been investigated for their association with dried vegetables. A single study assessed the prevalence of *Clostridium botulinum* in 10‐ to 25‐g retail samples of dried mushrooms; no positives were detected in 48 samples (Malakar et al. 2013).

Nine (or 10—with Canada in 2023) RASFF notifications were found for *B. cereus* in dried herbs and spices; three from ground ginger, and single occurrences in other spices (carvi moulu [caraway], ground cinnamon, curry powder, lemon grass powder, paprika powder, and soumbara spice). Reported *B. cereus* counts ranged from 4.2 to 6.7 log CFU/g with the highest value reported in soumbara spice, which is produced by fermentation after boiling and cleaning African locust bean seeds (Kouamé et al. 2020), possibly permitting excessive microbial growth of contaminants if not handled adequately (e.g., controlling time, temperature, pH development). In 2024, two notifications in the RASFF portal were related to isolation of *C. perfringens* from ground cinnamon, where 0–2.3 log CFU/g were reported.

### 3.2 *Cronobacter* spp. Prevalence and Recall Data

Two studies (one in Poland and one in the Czech Republic) analyzed 58 25‐g samples for *Cronobacter* spp. with no findings reported from cinnamon, oregano, dried sweet peppers, potato powder, or dried and candied fruits (Table 2; Hochel et al. 2012; Berthold‐Pluta et al. 2021). Hochel et al. (2012) analyzed 10–15 samples (25 g) of each product for *Cronobacter* spp. The highest contamination rate detected was 40% in basil. Garbowska et al. (2015) analyzed 38 10‐g samples of basil, parsley, and tarragon for *Cronobacter* spp.; a prevalence of 24% was found. Considering the widespread nature of *Cronobacter* spp. in the environment (Lou et al. 2014), this is unsurprising. Notably, no RASFF notification data could be found over the timeframe analyzed (2020–2024). It is possible that these products are not routinely tested for *Cronobacter* spp. because it is considered to mainly pose a risk for infants under 6 months and especially newborns, premature infants, and others in neonatal intensive care units (Donaghy et al. 2025). These groups are not likely to consume the products studied here. To date, no outbreaks related to *Cronobacter* spp. have been reported for dried fruits, vegetables, herbs, or spices.

### 3.3 Shiga Toxin‐Producing *E. coli* Prevalence and Recall Data

The prevalence of STEC (including O157) in herbs and spices was reported in four studies published in EFSA journals. In 2011, STEC was not detected in 119 samples analyzed, whereas in 2018, three out of 429 (0.7%) samples of dried vegetables yielded positive results (EFSA and ECDC 2013, 2019). In 2019, two out of 685 (0.29%) samples were positive for STEC (EFSA and ECDC 2021). Dried fenugreek leaves were the subject of two RASFF STEC notifications in 2020.

### 3.4 Hepatitis A Prevalence and Recall Data

One study analyzed 30‐g samples of figs and dates for HAV using PCR. No positive results were reported among 91 fig samples whereas two of 185 date samples (1.1%) tested positive (Boxman et al. 2012). Dried tomatoes from Turkey gave rise to two RASFF notifications for Hepatitis A in 2020 and 2021. These notifications may reflect increased surveillance following Hepatitis A outbreaks in Europe linked to semi‐dried tomatoes in 2010 and 2011 (Table 1, Section 2).

### 3.5 *L. monocytogenes* Prevalence and Recall Data

No RASFF notifications were identified for *L. monocytogenes* contamination of dried fruits and vegetables. A study conducted in Wales did not detect *L. monocytogenes* at levels >100 CFU/g in any of 555 dried fruit samples (Table 2). Several studies have evaluated the prevalence of *L. monocytogenes* in herbs and spices; however, in studies reporting quantitative data, all levels were <100 CFU/g. Across these investigations, sample sizes varied (up to 25 g), and of the 2,164 samples analyzed, 26 (1.2%) were positive for *L. monocytogenes*. Because analytical sample sizes and study designs differed, prevalence estimates and quantitative data cannot be directly compared. The highest reported prevalence (17/282; 6.0%) was documented in 2014, although the analytical sample size was not specified. Between 2020 and 2024 no RASFF notifications or recalls were identified for *L. monocytogenes* in dried herbs and spices.

### 3.6 *Salmonella* Prevalence and Recall Data

*Salmonella* was not detected in 555 25‐g samples of dried fruits collected in Wales (Meldrum et al. 2006) but was isolated from 37 of 70 (52%) samples (25 g) of sun‐dried tomatoes and 20 of 70 (28%) samples of raisins collected from public markets in Mexico (Table 2; Aguilar‐Vázquez et al. 2022). In several studies, attempts were made to quantify *Salmonella* through enumeration on selective media (usually brilliant green agar). In these studies, *Salmonella* was not detected above the level of detection (LOD) (<10 CFU/g) in 164 commercial dried fruits, 40, 20, or 15 home dried apples, peaches, or spinach, respectively (Ntuli et al. 2017). However, levels of 1.0–1.6 log CFU/g were reported in raisins and prunes (South Africa; Witthuhn et al. 2005), 1.0–1.5 log CFU/g in dried vegetable leaves (Lesotho; Ntuli et al. 2017) and 1.0–4.7 log CFU/g in raisins (India; Sharma et al. 2008). In the RASFF portal, 10 notifications related to *Salmonella* were found within the time interval reviewed (2020–2024); one each for banana chip, black mulberry, raisins, and tomato flakes, two for dried mushrooms, and four for dried onions.

The prevalence of *Salmonella* in herbs and spices was reported in several studies (Table 2); however, analytical sample sizes were not specified in all cases, complicating direct comparison of the findings. In EFSA and ECDC publications, analytical units were typically 25‐g samples. The highest reported prevalence was 5.8%, whereas the lowest was 0%, in both herbs and spices.

Although the US FDA risk profile focused on samples collected at ports of entry (US FDA 2017), Zhang et al. (2017) studied the *Salmonella* prevalence at retail in the US. In that study, *Salmonella* prevalence ranged from 0% to 0.64%, substantially lower than rates reported at ports of entry (US FDA 2017). A prevalence of 0.64% was observed for red pepper, whereas *Salmonella* was not detected in cumin or white pepper (Zhang et al. 2017). Differences in reported prevalence may reflect treatments applied by manufacturers in the US; however, analytical sample sizes also differed between the studies (125 g in Zhang et al. 2017 vs. 750 g in US FDA 2017).

RASFF data from 2020 to 2024 included 250 notifications for black pepper, representing 79% of all *Salmonella* notifications (only qualitative data, i.e., presence reported); 17 of these notifications (5.4%) involved paprika. Additional notifications were reported for coriander and cumin (four each), chili, fenugreek leaves, and ginger (three each), basil, dill, mint leaves, and turmeric (two each), and single notifications of *Bixa orellana* seeds, laurel, marjoram, mustard seeds, nigella seeds, and oregano. Ten notifications involved spice mixes, including three curry powders for which the specific contaminated component could not be identified.

### 3.7 *Staphylococcus aureus* Prevalence, Levels, and Recall Data

No RASFF notifications related to *S. aureus* and dried fruits and vegetables were published between 2020 and 2024. Three studies investigated *S. aureus* populations in dried fruits and vegetables using direct plate‐counting methods (Table 2). In a study conducted in Wales, none of 555 dried fruit samples exceeded a threshold of ≥2 log CFU/g of *S. aureus* (Meldrum et al. 2006). In India, *S. aureus* was not found on 20 raisin samples (LOD >2 log CFU/g) (Sharma et al. 2008). Similarly, a study in South Africa did not detect *S. aureus* in three samples each of dried apricots and prunes, or four samples each of dried nectarines and peaches; however, the organism was detected in three raisin samples at 10, 10, and 20 CFU/g (Witthuhn et al. 2005).

## 4 Pre‐ and Postharvest Activities

The safety of dried plant materials is built on the foundation of prevention or reduction of contamination and proliferation of pathogens during pre‐ and postharvest activities. Pre‐harvest contamination of the raw material is a potential source of pathogens in dried ingredients if not prevented or reduced. The raw material pathogen load is an input to the risk assessment of the finished dried material, considering the risk reduction delivered by the drying method and other processes. Dried ingredient manufacturers should have clear hygiene requirements for the farms and/or suppliers from which they source raw materials. Good Agricultural Practices (GAPs) should be practiced by the source farms. GAPs include the control of potential contamination sources such as the growing field and adjacent land, animal activity, manure‐based fertilizer, agricultural water source and treatment, plant disease or damage, human health and hygiene, harvesting practices, and equipment (Iwu and Okoh 2019; USDA 2024; US FDA 1998). Climate‐ and weather‐related factors, such as temperature, humidity, flooding, and wind, also impact the survival and dissemination of foodborne pathogens (Balta et al. 2024). Higher temperatures may increase the prevalence of pathogens in the environment, and heavy rainfall events may promote the survival and potentially loads of pathogens and the spread of bacteria via contaminated water (Duchenne‐Moutien and Neetoo 2021).

Postharvest handling, storage, and processing can increase or decrease pathogen risk. Routine cleaning, sanitation, and inspection of storage areas, packing facilities, and transportation containers help control cross‐contamination from previous materials. Storage areas should be dry and well ventilated. Washing and fluming of materials removes soil and some microorganisms but can also be a source of cross‐contamination if water quality is not maintained properly (López‐Gálvez et al. 2021). Washing equipment varies by commodity and investment level, with some machines including scrubbing with brushes, whereas others rely on water flow or agitation to mechanically remove soil. Delicate materials, such as blackberries, raspberries, or leafy herbs, are not conducive to abrasive washing methods. Sanitizers, such as hypochlorite or peracetic acid, enhance the microbial reduction of the wash or flume water (Banach et al. 2015). Peeling, cutting, and slicing can internally introduce microorganisms from the plant exterior or from equipment (Yousuf et al. 2020). Other postharvest treatments for quality purposes include sugar infusion, soaking, or fermentation. Sugar infusion is often used to sweeten dried fruits. However, it also causes osmotic dehydration that can reduce the overall drying time and improve the final product quality. Water or alkaline (lye) soaking, steaming, or fermentation may be used to facilitate peeling in some cases, such as for garlic, ginger, and white pepper (Prakash and Prasad 2023; Srikaeo et al. 2011; Zhang, Yu, et al. 2024). Pathogen inactivation during drying (with and without pre‐drying treatments), storage, and subsequent treatments is addressed in the following sections.

## 5 (Pre‐)drying Methods

Drying fruits, vegetables, herbs, and spices is a widely applied preservation strategy that effectively extends shelf life and increases microbial food safety. However, the degree of pathogen reduction achieved varies considerably (Table 3). This variability arises from differences in drying technologies, species and strain‐specific microbial responses, and physiological state of the cells. Pre‐drying interventions, commonly applied to prevent browning or texture loss, can also reduce microbial loads. Their efficacy depends on concentration, contact time, temperature, and the specific microorganism targeted.

**TABLE 3:** Effect of pre‐drying treatments and drying conditions on foodborne pathogen levels in dried fruits, vegetables, herbs, and spices.

Drying condition and matrix	Form	Inoculation method	Inoculum (no. of strains)	Pre‐drying treatment	Log reduction (pre‐treatment alone)	Drying conditions	Total log reduction (pre‐treatment + drying)	Comments	Reference
Conventional air drying									
Blue‐ berries	Whole	Broth culture, spot inoculation	*Salmonella* (3); *Escherichia coli* O157:H7 (3); *Listeria monocytogenes* (3); Hepatitis A virus (1)	Osmotic dehydration in sucrose solution (75 %, w/v) for 15 h, 23°C	1.5; 1.0; 3.9; 0.4	100°C, 1 h	>7.1; >6.8; >6.4; 2.6		Bai et al. (2020)
Chili peppers, red	Halved	Broth culture, spot inoculation	*S*. Typhimurium (1)	Air; ClO_2_ gas (Exposure at 25°C, 0.5–6 h)	0; >5 (after 0.5 h)	55°C, up to 24 h	4.5 (after 6 h air exposure), >5.8 after 24 h	Naturally occurring microbiota remain viable after gas treatment and drying at 55°C for 24 h.	Lee et al. (2018)
Convection air drying									
Apples	Sliced (cored) cv. Gala	Broth culture, surface inoculation (both sides)	*L. monocytogenes* (8)	None	0	60°C for up to 180 min or 80°C for up to 150 min, and air velocity at 0.7 or 2.1 m/s	0.6 or 1.3 (60°C, 120 min at 0.7 or 2.1 m/s); 1.8 or 2.8 (60°C, 180 min at 0.7 or 2.1 m/s); 1.7 or 3.6 (80°C, 90 min at 0.7 or 2.1 m/s); 5.2 (80°C, 150 min at both air velocities)	Greater *L. monocytogenes* inactivation during drying was achieved at higher temperature or higher air velocity.	Randriamiarintsoa et al. (2024)
Apples	Sliced (cored), 6 cultivars	Cell lawns, surface inoculation (both sides)	*Salmonella* (5)	Dipping in 0.5% citric acid, ascorbic acid, propionic acid, lactic acid, fumaric acid, sodium bisulfate, sodium benzoate, potassium sorbate	Not measured	Consumer‐grade food dehydrator set at 60°C for 5 h; racks rotated after 2.5 h	1.7–3.8: highest reduction achieved with pre‐treatment with fumaric>citric >acetic acid	Drying inactivation of *Salmonella* cocktail was dependent on apple cultivar possibly due to differences in pH.	Gurtler et al. (2020)
Apples	Sliced (peeled, cored) cv. Gala	Broth culture, immersion	*E. coli* O157:H7 (4)	None; Steam blanching (88°C, 3 min); Ascorbic acid (3.4%), 15 min	0; <1.0; 1.4‐1.6	57.2°C | or 62.8°C for 6 h	2.9 | 3.5; 2.0 | 3.6; 8.0 | 8.3	Log reductions on TSA and SMAC show similar results.	Burnham et al. (2001)
Apples	Sliced (peeled, cored) cv. Gala	Broth culture, immersion	*E. coli* O157:H7 (3)	10‐min: None; Water; Ascorbic acid (2.8%); Citric acid (1.7%); Lemon juice (50%); Lemon juice (50%) with preservatives	0; 0.9; 1.3; 1.3; 1.2; 0.9	62.8°C, 6 h	3.1; 5.1; 5.1; 6.7; 7.1; 6.7	Log reductions on TSA and SMAC show similar results. Dipping in acid solutions inhibits growth for 6 h at room temperature.	Derrickson‐Tharrington et al. (2005)
Apples	Sliced (peeled, cored) cv. Gala	Broth culture, surface inoculation (both sides)	*Salmonella* (5)	10‐min: None; Water; Sodium metabisulfite (4.2%); Ascorbic acid (3.4%); Citric acid (0.21%)	0; 0.2; 0.4; 0.7; 0.9	60°C, 6 h	2.8; 2.7; 4.3; 5.2; 3.8	Log reductions on TSAP and BGSA show similar results. Significantly larger log reductions were found on XLT4	DiPersio et al. (2003)
Apples	Diced (6.4‐mm cubes) cv. Gala	Cell lawn suspension applied to cubes	*Salmonella* (5); *E. faecium* (1)	None	NA	104°C for up to 2 h; 135°C for up to 2 h	*Salmonella* >6.8 (120 min), *E. faecium* 3.7 (120 min); *Salmonella* >7 (85 min), *E. faecium* 7 (95 min)	The top of 9‐cm bed depth (reductions provided) was farthest from heat source and bottom was closer. Significant reduction only seen >10 min at bottom and >40 min at top cubes	Grasso‐Kelley et al. (2021)
Apples	Sliced (cored) cv. Gala	Cell lawns suspensions on one side of slices	*Salmonella* (5)	2‐min immersion: water; sodium bisulfate (0.3%); e‐polylysine (0.1%); peracetic acid (42 ppm)	Not reported	60°C, 5 h	2.6; 6.2; 3.9; 5.6	Lower concentrations of the pre‐treatment solutions showed lower log reductions.	Gurtler et al. (2024)
Apples	Diced (6.4‐mm cubes) cv. Gala	Cell lawn suspensions applied to cubes by shaking	*Salmonella* (3)	High humidity air heating 90°C (9 min), 70°C (16 min); ascorbic acid (3.4%) 65°C (90 s)	6.2, 6.9; 7.9	90°C, 70 min	Not determined	High relative humidity created by moisture from apples enhances inactivation	Zhang, Yang, et al. (2024)
Apples	Diced cubes (≈6.4 mm) cv. Gala	Cell lawn suspensions applied to cubes by shaking;	*Salmonella* (5)	None	NA	88–120°C; air velocity 2.1–3.8 m/s; bed depth 5.1–12.7 cm	≥5 log (except low T, high bed depth, medium air velocity only 4.6	Non‐linear inactivation, T increase thrives inactivation.	Liu et al. (2025)
Basil	Leaves, whole	Broth culture spot inoculation, cell lawn for pilot plant drying	*Salmonella* (3); *L. monocytogenes* (1); *E. coli* O157:H7 (1); *E. coli* P1 (1); *L. innocua* (1); *E. faecium* (1)	None	NA	60°C | 80°C | or 100°C for 5‐120 min	*Salmonella*: 4.2 (90 min) | 6.2 (80 min) | 6.2 (30 min)	*S*. Senftenberg, most heat resistance pathogen, *E. coli* P1 showed similar resistance, *E. faecium* more resistant, pilot plant drying achieved less reduction when using 60°C, 90 min lab‐scale comparison	Zhou et al. (2020)
Cabbage	Sliced	Broth culture, immersion	*Salmonella* (1)	None	NA	50°C, 60°C, 70°C	3 (19.8 h, 4.4 h, 2.2 h)		Hawaree et al. (2009)
Cabbage	Sliced	Broth culture, immersion	*Salmonella* (1)	5‐min immersion: none; 0.5% acetic acid; 1.0% acetic acid; 1.5% acetic acid	0; 1.4; 1.5; 1.5	50°C | 55°C | 60°C	3 (22 | 4 | 2.1; 7.3 | 3.4 | 1.7; 8.1 | 3 | 1.6; 6.4 | 3.2 | 1.3)	Additional timepoints are described in the reference	Chiewchan and Morakotjinda (2009)
Cabbage	Sliced	Broth culture, immersion	*Salmonella* (1)	None; 0.5% acetic acid, 5 min; blanching, boiling water, 4 min; steam blanching, 2 min	0; 1.6; 3.8; 3.6	60°C using hot air	3 (270 min; 206 min; 124 min; 119 min)	Additional timepoints are described in the reference	Phungamngoen et al. (2013)
Cabbage	Sliced	Broth culture, immersion	*Salmonella* (1)	None	NA	50°C, 60°C, 70°C using hot air	3 (6.1 h, 3.3 h, 1.8 h)	Additional timepoints are described in the reference	Phungamngoen et al. (2011b)
Carrots	Sliced (peeled)	Broth culture applied to both sides of slices	*Salmonella* (5)	None; Steam blanching (88°C, 10 min); water blanching (88°C, 4 min); blanching in 0.105% citric acid (88°C, 4 min); blanching in 0.21% citric acid (88°C, 4 min)	0; 3.8; 4.6; 4.6; 4.2	60°C, 6 h	1.7; 4.0; 4.1; 4.9; 5.1	Log reductions on TSAP are shown. Slightly larger log reductions on XLD	DiPersio et al. (2007)
Carrots	Sticks (peeled and cut)	Broth culture, immersion	*Salmonella* (1)	None	NA	60°C	4 (11.7 h, smooth surface); 14 h, roughest surface)	Different cut surface types	Phungamngoen et al. (2011a)
Ginger root	Sliced	Cell lawn suspension applied onto slices	*Salmonella* (4)	NA	NA	51°C, 60°C	1 (24 h, lab oven), 2 (24 h, lab oven)	Survival related to rate of reduction in a_w_, and was tested in different oven types.	Gradl et al. (2015)
Green onions	Cut pieces (1.9 cm)	Inoculum spread on pieces, then dried	Hepatitis A	None	NA	50, 55, 60, or 65°C for 20 h	1.3, 2.0, 2.7, or 3.4	Values were calculated from linear regression presented in the reference.	Laird et al. (2011)
Mushrooms (portobello)	Sliced	Broth culture applied to slices	*S*. Typhimurium (3); *B. cereus* (3); *L. monocytogenes* (3)	None	NA	∼45°C, 8 h	2.5; 1.2; 2.5	Log reductions on TSA are shown. Slightly larger log reductions were found on selective media	Kragh et al. (2022)
Mushrooms (enoki and wood ear)	Cubed (2.5 cm)	broth culture applied by spot inoculation	*L. monocytogenes* (4); *Salmonella* (4)	None	NA	70, 80, or 90°C up to 24 h	4 (enoki): *L. monocytogenes* 24 h (70°C), 14.1 h (80°C, 3.1 h (90°C); *Salmonella* 9.1 h (80°C), 2 h (90°C).	Additional results are reported: Tailing effect for enoki at 70°C and 80°C. Reduction values in wood ear described in reference.	Salazar et al. (2023)
Peaches	Sliced	Broth culture applied to slices	*L. monocytogenes* (5)	10‐min immersion: none; water; sodium meta‐bisulfite (4.18%); ascorbic acid (3.40%); citric acid (0.21%)	0; 0.7; 1.5; 0.7; 0.5	60°C, 6 h	3.15; 3.15; 4.3; 5.1; 4.5	Log reductions TSAP are shown; slightly larger on PALCAM	DiPersio et al. (2004)
Peaches	Halved	Cell lawn suspension spot inoculated to halves	*Salmonella* (5); *E. faecium* (1)	None; Water, 10 min; syrup blanching (5 min); ascorbic acid (∼40 g/L), 10 min; citric acid (∼5.3 g/L), 10 min; lemon juice (50%), 10 min; sodium metabisulfite (22 g/L), 10 min	*Salmonella* 0; 0.1; <6.7; 0.1; 0.1; 2.5; 0.1	60°C, 72 h	No pre‐treatment: *Salmonella* 1.5; *E. faecium* 2.0	Log reductions on TSAR are shown. Sun drying mimicked achieving >6 log reduction (21 days).	Liu, Sheng et al. (2022)
Tomatoes (Roma)	Halved	Broth culture, spread on cut surface	*Salmonella* (5)	10‐min immersion: none; water; ascorbic acid (AA) (3.40%); citric acid (0.21%)	0; 1.0; 1.1; 1.3	60°C, 14 h	3.2; 3.8; >6.0; 5.1 (unpeeled tomatoes)	Peeled tomato results similar; increased log reductions by acid treatment before drying	Yoon et al. (2004)
Freeze drying									
Coriander	Leaves, stems (2‐cm pieces)	Broth culture, spot inoculation	*E. coli* O157:H7 (3); *L. monocytogenes* (3); *Salmonella* (3)	Freezing at −80°C overnight	0.5–0.8; 0.2; 0.7–0.8	−55°C (0.012 bar for 23.5 h, then 0.007 bar for 30 min)	1.4–1.5; 0.7; 1.9–2.0		Bourdoux et al. (2018)
Low‐pressure superheated steam drying									
Cabbage	Sliced	Broth culture, immersion	*Salmonella* (1)	None; acetic acid (0.5%), 5 min; water blanching, 4 min; steam blanching, 2 min	0; 1.6; 3.8; 3.6	60°C and 10 kPa, 60 min	3 (58 min; 80 min; 44 min; 48 min)	Additional timepoints are described in the reference	Phungamngoen et al. (2013)
Cabbage	Sliced	Broth culture, immersion	*Salmonella* (1)	None	NA	50, 60, or 70°C	3 (2.1, 0.8, or 0.4 h)	Additional timepoints are described in the reference	Phungamngoen et al. (2011b)
Supercritical (sc) CO_2_ drying									
Coriander	Leaves, stems (2‐cm pieces)	Broth culture, spot inoculation	*E. coli* O157:H7 (3); *L. monocytogenes* (3); *Salmonella* (3)	None	NA	scCO_2_ of 80 bar at 35°C, 150 min of drying	5.6–5.7; 6.7; 5.9–6.1	No thermal effect since water is not removed by vaporization but is dissolved in scCO_2_. Without drying 4‐5 log reductions achieved	Bourdoux et al. (2018)
Apple	Slices	Broth culture, spot inoculation	*E. coli* O157:H7 (3); *L. monocytogenes* (3); *Salmonella* (3)	None	NA	Sc‐CO_2_ at 100 bar, 40°C, 10 min pressurization, and 20 min depressurization	5; 7; 5		Zambon et al. (2021)
Strawberry	Slices	Broth culture, spot inoculation	*E. coli* O157:H7 (3); *L. monocytogenes* (3); *Salmonella* (3)	None	NA	Pressurization to 5 MPa, then to 10 MPa, and then depressurization Internal temperature 40°C	5; 7; 5		Zambon et al. (2022).
Vacuum drying									
Cabbage	Sliced	Broth culture, immersion	*Salmonella* (1)	None; acetic acid (0.5%), 5 min; blanching, boiling water, 4 min; steam blanching, 2 min	0; 1.6; 3.8; 3.6	60°C and 10 kPa, 60 min	3 (94 min; 88 min; 52 min; 71 min	Additional timepoints are described in the reference	Phungamngoen et al. (2013)
Cabbage	Sliced	Broth culture, immersion	*Salmonella* (1)	None	NA	50, 60, or 70°C and 10 kPa for up to 2 h	3 (2.4, 1.4, or 1.1 h)	Additional timepoints are described in the reference	Phungamngoen et al. (2011b)

Abbreviations: BGSA, brilliant green sulfa agar; LOD, level of detection; NA, not applicable; PALCAM, Listeria selective agar; SMAC,

sorbitol MacConkey agar; TSA, tryptic soy agar; TSAP, tryptic soy agar with 0.1% pyruvate; TSAR, tryptic soy agar with rifampicin at

50 mg/mL; XLT4, xylose lysine tergitol 4

### 5.1 Air Drying

Air drying is among the oldest preservation methods, but traditional open‐air practices are highly vulnerable to contamination from dust, insects, and variable climatic conditions, compromising both safety and quality. On‐farm approaches, such as multi‐day sun drying or cabinet (tray) drying, are still widely applied but provide limited hygienic control. In contrast, convective air drying offers a more controlled alternative, circulating warm, dry air around the product to gradually reduce moisture while minimizing thermal damage. Although this method can reduce microbial loads, process parameters are typically optimized for product quality rather than pathogen inactivation. Traditional sun drying exemplifies this limitation, as environmental exposure makes control of process parameters impossible and creates opportunities for contamination. These vulnerabilities persist during subsequent transport and storage, especially across countries with variable hygiene standards. Consequently, in importing regions, additional decontamination steps are often necessary to ensure microbial safety.

### 5.2 Thermal Drying

In general, convective hot‐air drying relies on generated and forced hot air and differs from other drying processes by combining moisture removal with a mild thermal inactivation effect to extend shelf life. As dehydration itself does not guarantee pathogen inactivation, the heat applied during many drying processes acts as a crucial hurdle. Evidence indicates that survival depends strongly on drying temperature. More generally, higher drying temperatures accelerate microbial inactivation, but conditions are usually intended primarily for water removal and shelf stability and optimized for preserving sensory quality rather than eliminating pathogens. Most studies still focus on reductions in native microbial populations or report presence/absence data rather than direct inactivation of inoculated pathogens. Evidence from different commodities illustrates common patterns and critical variables that govern pathogen inactivation during thermal drying (Table 3). Across apple, basil, ginger, and pepper, a rapid initial decline in microbial counts is consistently followed by slower inactivation as products become progressively drier, reflecting the protective effect of reduced water activity on microbial survival (see, e.g., Gradl et al. 2015; Lee et al. 2018; Liu et al. 2025; Randriamiarintsoa et al. 2024; Zhou et al. 2020). This nonlinear inactivation highlights the challenges in achieving adequate pathogen reduction with drying alone.

*Salmonella* Senftenberg showed the greatest heat resistance among the pathogens tested on basil (*Salmonella* Senftenberg, *Salmonella* Enteritidis, *Salmonella* Thompson, *L. monocytogenes*, and *E. coli* O157:H7), yet substantial reductions were still achieved at progressively higher drying temperatures (e.g., >4‐log reduction after 90, 40, and 20 min at 60, 80, and 100°C, respectively) (Table 3; Zhou et al. 2020). Reduced water activity during dynamic drying further increased thermal resistance, highlighting the critical role of product moisture status. Pilot‐scale trials with surrogate strains (e.g., *Enterococcus faecium, E. coli* P1, and *Listeria innocua*) validated the decontamination potential and underscored the importance of selecting suitable surrogates for process evaluation. Together, these findings demonstrate that thermal drying efficacy depends not only on temperature but also on the kinetics of water activity reduction and moisture content, both of which govern microbial survival and the robustness of pathogen control.

Pre‐drying treatments can have a significant impact on the microbial inactivation (Table 3). For instance, a study on carrot slices found that log reductions of *Salmonella* Typhimurium increased when blanching was applied prior to thermal drying (DiPersio et al. 2007). Pretreatment of apple slices with acidic or metabisulfite solutions resulted in enhanced inactivation of *Salmonella* during thermal drying (DiPersio et al. 2003). These studies illustrate that the drying process alone often exhibits diminishing lethality as moisture is lost, but combining pretreatments with thermal drying can overcome this limitation and achieve more reliable pathogen control.

Liu et al. (2025) further demonstrated that temperature, air velocity, and bed depth strongly influence inactivation dynamics: Higher temperatures and increased airflow accelerated heating and moisture loss, whereas thinner bed depths improved uniformity, collectively resulting in faster and greater *Salmonella* reductions. Slower airflow enhanced *Salmonella* inactivation in ginger (Gradl et al. 2015); higher air velocity increased lethality with apple slices (Liu et al. 2025). Randriamiarintsoa et al. (2024) reported similar trends for *L. monocytogenes* on apples dried at 60°C, with greater inactivation at air flows of 2.1 m/s than at 0.7 m/s. This contrast reflects commodity‐specific drying dynamics: in apples, higher airflow promotes a faster temperature increase by shortening the constant‐rate drying period, thereby increasing the exposure of microorganisms to lethal conditions earlier in the process. In contrast, in ginger, slower airflow prolongs surface moisture retention, that is, higher water activity retention, thereby leading to an increased lethal effect under those conditions (Gradl et al. 2015). These findings underscore that air velocity effects are strongly dependent on tissue structure, porosity, and moisture dynamics, and that process parameters, such as bed depth and airflow, should be validated for each commodity to ensure consistent microbial control. In addition, variety‐specific characteristics influenced outcomes. Gurtler et al. (2020) reported survival of desiccation‐resistant *Salmonella* on apples that was cultivar‐dependent, with Fuji, Granny Smith, and Pink Lady varieties supporting greater inactivation than Envy and Gala under identical drying conditions. Pretreatments further modulated outcomes: Sodium bisulfate and fumaric acid dips before dehydration achieved the most pronounced reductions, whereas other acids and preservatives provided intermediate effects. Comparable studies on cabbage and carrot slices support that both commodity traits and chemical pretreatments strongly shape microbial survival during convective air drying (Phungamngoen et al. 2011b, DiPersio et al. 2007).

### 5.3 Osmotic Dehydration

Osmotic dehydration is widely applied to fruits to reduce moisture and preserve quality; however, its microbiological efficacy depends strongly on process temperature and on whether it is combined with additional treatments. Bai et al. (2020) investigated blueberries subjected to osmotic dehydration at different temperatures, as well as a combined process involving cold infusion (osmotic dehydration at 23°C) followed by hot‐air drying at 100°C. *E. coli* O157:H7, *L. monocytogenes*, *S. enterica*, and HAV, were studied alongside surrogates *E. faecium*, *E. coli* P1, *L. innocua*, murine norovirus (MNV), and bacteriophage MS2. Osmotic dehydration at 23°C achieved 1.0–3.9 log reductions of bacterial pathogens on blueberries, whereas *E. faecium* was the most resistant surrogate. Populations of viruses HAV and MNV declined by 0.4 and 0.2 log, respectively. When combined with subsequent hot‐air drying at 100°C all bacterial strains were reduced by >6 log, and both HAV and MNV were substantially decreased, with 2.6 and >3.4 log reduction, respectively. These findings underscore that osmotic dehydration alone can control bacterial pathogens to a certain extent, whereas the combination of osmotic dehydration with subsequent thermal drying markedly enhances microbial inactivation, ensuring the control of bacterial pathogens while achieving meaningful reductions in enteric viruses.

### 5.4 Supercritical CO_2_ Drying

Supercritical CO_2_ (scCO_2_) drying has emerged as a promising low‐temperature technology that couples dehydration with microbial inactivation. Unlike conventional drying methods, scCO_2_ operates under high pressure and moderate temperature, enabling water removal, while simultaneously inactivating pathogens. Bourdoux et al. (2018) demonstrated the efficacy of scCO_2_ drying of coriander leaves inoculated with *E. coli* O157:H7, *L. monocytogenes*, or *S. enterica*. Treatments at 80 bar and 35°C achieved >5 log reductions of all pathogens, clearly outperforming freeze‐drying, which produced only limited inactivation. Populations of natural yeasts, molds, and Enterobacteriaceae were reduced during treatment; however, bacterial spores were able to survive the treatment.

Comparable findings were obtained for fruits. Zambon et al. (2022) showed that scCO_2_ drying of strawberry slices at 10 MPa and 40°C removed nearly all water within 6 h while reducing inoculated populations of *E. coli* O157:H7, *L. monocytogenes*, and *Salmonella* spp. by >5 log. Natural mesophilic bacteria were less susceptible, indicating some selectivity in microbial response. Similarly, Zambon et al. (2021) confirmed on apple slices that scCO_2_ drying at 100 bar and 40°C achieved ∼5‐log reduction of *E. coli* O157:H7, and *Salmonella* spp., and ∼7 log reduction of *L. monocytogenes*, reducing all pathogens below enrichment limit of detection, immediately after pressurization and depressurization. Natural microflora, including yeasts and molds, were also efficiently reduced, whereas spores persisted across treatments. Importantly, the microbiological stability of scCO_2_‐dried apples was maintained for up to 12 months. Together, these studies highlight the potential of scCO_2_ drying as a hybrid process that combines drying and pasteurization in a single step. Its advantages include strong pathogen inactivation at mild temperatures. Limitations include incomplete control over bacterial spore‐formers and variable efficacy against background microbiota, pointing to the need for possible integration with additional hurdles.

### 5.5 Vacuum Drying

Vacuum drying accelerates moisture removal due to reduced boiling temperatures under lower pressures, enabling faster drying while better preserving heat‐sensitive product quality. However, vacuum drying may not achieve adequate microbial inactivation because bacterial destruction depends not only on dehydration but also on sufficient temperature–time exposure. In contrast, low‐pressure superheated steam drying enhances microbial inactivation through rapid surface heating and steam condensation, which promotes early thermal lethality. Consequently, low‐pressure superheated steam drying generally shows greater effectiveness in reducing pathogens such as *Salmonella* compared with vacuum drying, although both methods improve drying efficiency compared with conventional hot air drying (Phungamngoen et al. 2013).

## 6 Pathogen Survival in Dried Matrices

Numerous studies have examined the short‐ and long‐term survival of pathogens on dried fruits, vegetables, herbs, and spices to understand the consequences of pathogen contamination on products that are dried and sold to consumers as RTE (Table 4). Dried products can be stored at ambient temperatures due to the microbial and chemical stability provided by their low water activity. However, these products may also be stored or transported under a wide range of conditions from cold (e.g., refrigerated) to elevated temperatures (>30°C). Pathogens survive better at reduced temperatures compared to ambient or elevated temperatures, independent of the type of dried fruit, vegetable, herb, or spice (Table 4). Extended survival times at reduced temperatures were observed for *Salmonella* on dried cranberries, raisins, and strawberries (Beuchat and Mann 2014), on dates and dried pluots (Canakapalli et al. 2022), and dried apples (Jayeola et al. 2022). Similar results were observed for *E. coli* O157:H7 on dates and dried pluots (Canakapalli et al. 2022), and for *L. monocytogenes* on dried apples, raisins, and strawberries (Cuzzi et al. 2021), dates, and dried pluots (Canakapolli et al. 2022). Likewise, on dried vegetables (e.g., tomatoes), herbs, and spices, lower storage temperatures resulted in enhanced pathogen survival. Generally, cold storage resulted in slowest rates of pathogen reduction, followed by storage at ambient temperatures (Canakapalli et al. 2022; Li et al. 2020). Some studies investigated the impact of higher storage temperatures (e.g., 35°C, 37°C) and reported greater reductions at these temperatures when compared to those observed at ambient temperatures (Gradl et al. 2015; Keller et al. 2013; Ristori et al. 2007).

**TABLE 4:** Storage survival studies of foodborne pathogens on dried fruits, vegetables, herbs, and spices.

Pathogen and matrix, form	Water activity (a_w_)/pH	Pretreatment	Inoculum	Storage conditions	Storage time	Reduction counts	Comments	Reference
*Bacillus cereus*								
Mushrooms (portobello), sliced	a_w_ 0.17	Inoculated and dried in dehydrator (45°C, 8 h)	Strains (3); broth culture suspended in PS; applied to sample and hand massaged	Room temp (undefined), vacuum packaged	56 days	1.2 log (drying), <1 log (storage)	Inoculated at 4.5 log CFU/g. Soaking overnight at ambient temp led to population increases to 7–8 log CFU/g.	Kragh et al. (2022)
*Bacillus cereus* (spores)								
Spices, herbs	a_w_ 0.38 (parsley) to 0.47 (basil)	Steam, none	Strains (3); suspension used to inoculate sand	Ambient (24 ± 2°C), dark	50 weeks	Insignificant (≤1 log)	Allspice, basil, cinnamon, nutmeg, oregano, paprika, parsley, pepper	Dinh Thanh et al. (2018)
*Escherichia coli* O157:H7								
Dates (Medjool), whole	a_w_ 0.55–0.62, pH 5.6–5.8	None	Rifampin‐resistant strains (5); agar lawn on TSA (rif), suspended in PBS to inoculate sand	5, 20°C	180 days	>5.5 log at 180 days (5°C) and 90 days (20°C)	Initial populations ∼7 log CFU/g after drying. No difference low and high moisture dates. Not detected in 25‐g samples at day 90 (20°C) or day 180 (5°C).	Canakapalli et al. (2022)
Pluots, sundried halves	a_w_ 0.68, pH 3.4	SO_2_	Rifampin‐resistant strains (5); agar lawn on TSA (rif), suspended in PBS	5, 20°C	180 days	>4 log at 15 days at 5 and 20°C	Initial populations ∼6 log CFU/g after drying. Not detected in 25‐g samples at day 15 (5 and 20°C).	Canakapalli et al. (2022)
Tomatoes, sundried halves	a_w_ 0.78, pH 3.8	None	Rifampin‐resistant strains (5); agar lawn on TSA (rif), suspended in PBS	5, 20°C	180 days	>6 log at day 60 (5°C) or day 30 (20°C)	Initial populations ∼9 log CFU/g after drying. Not detected in 25‐g samples at day 30 (20°C) or day 60 (5°C).	Canakapalli et al. (2022)
*Escherichia coli* (STEC)								
Apricots, whole	With SO_2_, a_w_ 0.72, pH 3.5; without SO_2_, a_w_ 0.62, pH 4.3	With or without SO_2_	Strains (7); agar lawn, liquid suspension or dried on sand prior to inoculation	Ambient (undefined)	90 days	With liquid inoculum, ∼3.5 log at ≥30 days without SO_2_; not detected in 6×25 g ≥30 days with SO_2_	Survival was better with wet inoculum carrier, greater survival on fruit without SO_2_, indicating the antimicrobial properties of SO_2_.	Liu et al. (2021)
*Listeria monocytogenes*								
Apples, diced	a_w_ 0.4–0.6	“Natural”	Strains (4); grown on TSAYE, sand (SiO_2_) inoculated with lawn, dried and used as carrier	4°C, 25–81% RH; 23°C, 30–35% RH	0 days (study lasted 336 days)	>6.4 log on day 0	Target inoculum 6.8 log CFU/g before drying. Could not be recovered from the dried apples after inoculation (day 0).	Cuzzi et al. (2021)
Apricots, whole	With SO_2_, a_w_ 0.72, pH 3.5; without SO_2_, a_w_ 0.62, pH 4.3	With or without SO_2_	Strains (5); agar lawn, liquid suspension or dried on sand prior to inoculation	Ambient (undefined)	90 days	With liquid inoculum: ∼3.5 log at ≥15 days without SO_2_; with SO_2_ detected in 25 g at 60 days (0/6) and 90 days (1/6)	Survival better with wet inoculum carrier, greater survival on fruit without SO_2_, indicating the antimicrobial properties of free SO_2_.	Liu et al. (2021)
Black peppercorns	a_w_ 0.40	None	Rifampin‐resistant strains (4); cell lawns suspended in BPB	25°C at 25, 45, or 75% RH	180 days	6.3 to >9 log	Lower reductions at lower RH. Rate of reduction: ‐0.036, ‐0.047, and ‐0.058 log CFU/g per day at 25, 45, and 75% RH.	Salazar et al. (2019)
Dates (Medjool), whole	a_w_ 0.55–0.62, pH 5.6–5.8	None	Rifampin‐resistant strains (5); agar lawn on TSA (rif), suspended in PBS to inoculate sand	5, 20°C	180 days	>4 log in 150 days at 5°C and in 90 days at 20°C	Initial populations 6.2‐6.6 log CFU/g after drying. Better survival in low moisture dates. Not detected in 25‐g samples at day 90‐120 (20°C) or ∼day 150 (5°C).	Canakapalli et al. (2022)
Mushrooms (portobello), sliced	a_w_ 0.17	Inoculated and then dried in home dehydrator at 45°C for 8 h	Strains (3); broth culture suspended in PS; applied to samples and hand massaged	Room temp (undefined), vacuum packaged	56 days	2.6 log from initial drying and additional 2 log after 56 days	Initial populations 7.2 log CFU/g. Soaking overnight at ambient temp led to population growth to 7–8 log CFU/g.	Kragh et al. (2022)
Mushrooms (enoki and wood ear), 2.5‐cm cubes	a_w_ 0.24–0.29	Dehydrated in laboratory at 60°C for 24 h before inoculation	Rifampin‐resistant strains (4); broth culture washed in BPB; dried 24 h after inoculation	25°C, 33% RH	180 days	Enoki: ∼1.0, 2.4, and >4.2 log (28, 90, and ≥120 days). Wood ear: ∼4 log (day 30)	Initial populations ∼6 log CFU/g. Enoki: 4‐log reduction (inactivation with a tail) at ∼160 days. Wood ear: ∼2 log decline during post‐inoculation drying	Fay et al. (2023)
Peaches (Redhaven, Glohaven), sliced	a_w_ <0.35–0.50, pH ∼4.2 (untreated)	Ascorbic acid, citric acid, or metabisulfite solutions; water and no treatment controls	Strains (5); broth culture, suspended in PBS	25°C, 30% RH	14 days	∼1–3 log	Slices immersed in treatment solutions (10 min) before dehydration (60°C, 6 h). Initial populations after drying were 4–5 log CFU/g for controls, and 1–2 log CFU/g for treatments. At 14 days, populations were 1.8 log (controls) 1.4 log (sodium metabisulfite) and <1.1 log CFU/g (ascorbic and citric acid).	DiPersio et al. (2004)
Pluots, sundried halves	a_w_ 0.68, pH 3.4	SO_2_	Rifampin‐resistant strains (5); agar lawn on TSA (rif), suspended in PBS	5, 20°C	180 days	>3 log in 15 days at 5 and 20°C	Initial populations ∼5 log CFU/g after drying. Not detected in 25‐g samples at day 15 (5 or 20°C).	Canakapalli et al. (2022)
Raisins (Thompson), whole jumbo	a_w_ ∼0.4–0.5, (pH not provided)	NS	Strains (4); grown on TSAYE, lawn used to inoculate sand (SiO_2_), dried and then used as carrier	4°C, 25–81% RH; 23°C, 30–35% RH	336 days	1.4 log after 336 days at 4°C and by >4.2 log after 14 days at 23°C	Initial populations 4.6 log CFU/g; decreased below LOD (0.4 log CFU/g) after 14 days at 23°C. Differential strain survival. Greater survival of 3a than 1/2a strain at 4°C.	Cuzzi et al. (2021)
Strawberries, whole	a_w_ ∼0.4–0.5, (pH not provided)	NS	Strains (4); grown on TSAYE, lawn used to inoculate sand (SiO_2_), dried and then used as carrier	4°C, 25–81% RH; 23°C, 30–35% RH	336 days	3.1 log after 336 days at 4°C and by >4 log after 7 days at 23°C	Initial populations 4.0 log CFU/g; decreased below LOD (0.4 log CFU/g) after 7 days at 23°C. Differential strain survival. Greater survival of 3a than 1/2a strain at 4°C.	Cuzzi et al. (2021)
Tomatoes, sundried halves	a_w_ 0.78, pH 3.8	None	Rifampin‐resistant strains (5); agar lawn on TSA (rif), suspended in PBS	5, 20°C	180 days	1 to 3 log at 30 days; >5 log at 60 days at 5 and 20°C	Initial populations ∼7–8 log CFU/g after drying. Not detected in 25‐g samples at day 60 at 5 and 20°C.	Canakapalli et al. (2022)
*Salmonella*								
Apples, 9×9×2 mm pieces, dehydrated	a_w_ 0.56, pH 4.5	NS	Strains (5); agar lawns; suspended in water; spot inoculation ∼9 log CFU/piece	4, 25°C	110 days	*>*7 log at 82 days (4°C) and 46 days (25°C)	*Salmonella* stored at 4 and 25°C did not form colonies on TSAYE (82 days) or TSA‐YEP (46 days), but confocal microscopy suggested a high percentage of viable *Salmonella* after those times	Jayeola et al. (2022)
Apples (Gala), sliced	a_w_ 0.20–0.22 (dried 6 h), pH 4.0–4.5 (dried 6 h, untreated)	Ascorbic acid, citric acid, or metabisulfite solutions; water and no treatment controls	Strains (5); broth culture, suspended in PBS	25°C	28 days	∼2 log (controls); ∼3 log (sodium metabisulfite and citric acid); >3.7 log (ascorbic acid)	Inoculated prior to treatment, drying and storage. Immersion in acidic or metabisulfite solutions (10 min) before dehydration (60°C, 6 h).	DiPersio et al. (2003)
Apricots, whole	With SO_2_, a_w_ 0.72, pH 3.5; without SO_2_, a_w_ 0.62, pH 4.3	With or without SO_2_	Serovars (5); agar lawn, liquid suspension or dried on sand prior to inoculation	Ambient (undefined)	90 days	5 to >7 log	Pathogens survived for longer times or at higher levels on fruit without SO_2_, indicating the antimicrobial properties of free SO_2_. Survival better when inoculated with sand.	Liu et al. (2021)
Black peppercorns	NS	None	Serovars (5); agar lawns, suspended in PBS, inoculated, air‐dried	4, 25°C, 20, 60% RH	14 days	0.7–4.2 log	No significant reduction at 4°C, independent of RH; greatest reduction at ambient temp and 20% RH. Temperature had more impact than RH. Significant serovar impact.	Li et al. (2020)
Black peppercorns	NS	Steam	Serovars (5), *E. faecium*, cell lawns suspended in BPW	∼23°C; a_w_ 0.45	15 days	1.2 log	Initial populations 7.6 log CFU/g, 0.9 log reduction after grinding and equilibration (2 days), <0.2 log decrease during storage. *E. faecium* more resistant than *Salmonella*.	Wei, Vasquez, et al. (2021)
Black pepper, ground	a_w_ 0.51‐0.59	Unknown	Serovars (4); cell lawns suspended in SPB	25 or 35°C at ambient (≤40%) or 97% RH	365 days	2.8 to >5.3 log	Initial populations 7–8 log CFU/g Greater reductions at higher temperature and higher RH. At 35°C and high RH: below detection limit (1.7 log) after 60 days. At 25°C and low RH, 3‐log reduction in 50 days.	Keller et al. (2013)
Black pepper, ground	a_w_ 0.65	None	*S*. Rubislaw (1); broth culture	5, 25, 35°C; a_w_ 0.663–0.937	2, 15 days	1.2 to >5.0 log	Reductions at 35°C >25°C >5°C; greatest reduction at 35°C and high a_w_. Most reduction in first 2 days of storage.	Ristori et al. (2007)
Black pepper, ground	a_w_ 0.53	None	*S*. Enteritidis PT 30 (1), other serovars (3), *E. faecium*; cell lawns suspended in BPW	21°C; a_w_ 0.3, 0.5	365 days	0.8–3.2 log	Initial populations 7‐8 log CFU/g. Lower reduction at lower a_w_. Slower decline of *E. faecium* compared with *Salmonella*.	Xie et al. (2022)
Carrots (Nantes), 0.5‐cm slices	a_w_ 0.33‐0.37	Steam blanching at 88°C (4 or 10 min), or with 0.10% or 0.21% citric acid solution at 88°C (4 min)	Serovars (5); broth culture, suspended in PBS	25°C	0–30 days	>0.7 to 1.4 log; undetectable by enrichment of sample in 0.21% citric acid‐treated product	Inoculated at 7.8 log CFU/g prior to treatment, drying at 60°C (6 h) and storage. At day 0, populations ranged from 5.2 log CFU/g (no treatment) to ∼3 log CFU/g for water/steam blanched to ∼2 log CFU/g for acid‐blanched carrots.	DiPersio et al. (2007)
Cinnamon, ground	a_w_ 0.51	None	*S*. Enteritidis PT 30 (1), other serovars (3), *E. faecium*; cell lawns suspended in BPW	21°C a_w_ 0.3, 0.5	365 days	4.3 to >6 log	Initial population ∼8 log CFU/g. Slower decline of *E. faecium* compared with *Salmonella*.	Xie et al. (2022)
Chili, ground	a_w_ 0.50	None	*S*. Enteritidis PT 30 (1), other serovars (3), *E. faecium*; cell lawns suspended in BPW	21°C a_w_ 0.3, 0.5	365 days	4.5 to >6 log	Initial populations 8–9 log CFU/g. Lower reduction at lower a_w_. Slower decline of *E. faecium* compared with *Salmonella*.	Xie et al. (2022)
Cranberries, whole, sweetened + other ingredients	a_w_ 0.47, pH 2.52	None	Serovars (5); broth suspended in DW or PW (mist or dried on sand)	4, 25°C	242 days	Mist: >4 or >5 log in 1 day at 4 or 25°C. Sand: >2 log in 1 or 84 days at 4 or 25°C	Initial populations 6.9 log (mist) and 3.4 log CFU/g (sand). Acid adapted cultures grown in 1% glucose added to TSB or TSA. Better survival when inoculated with dry sand than mist. Not detected in 25‐g samples: mist, 25°C, 42 days; sand, 25°C, 21 days. At 4°C some samples positive at 242 days (mist and sand).	Beuchat and Mann (2014)
Dates (Medjool), whole	a_w_ 0.55–0.62, pH 5.6–5.8	None	Rifampin‐resistant serovars (5); agar lawn on TSA (rif), suspended in PBS to inoculate sand	5, 20°C	180 days	1.6‐1.9 log at 5°C; >5 log at day 90 at 20°C	Initial populations 6 and 7 log CFU/g after drying. No difference between low or high moisture dates. Not detected in 25‐g samples at day 150 (20°C). Populations at ∼5 log CFU/g at day 180 at 5°C.	Canakapalli et al. (2022)
Ginger, ground	a_w_ 0.53	None	Serovars (4); cell lawns suspended in BPW	25, 37°C, 33, 97% RH	≤365 days	2.1 to >6.3 log	Initial populations 7 to 8 log CFU/g. Greater reductions at higher temperature or higher humidity (range: ‐0.01 to ‐1 log CFU/g/day).	Gradl et al. (2015)
Mushrooms (portobello), sliced	a_w_ 0.17	Dried in home dehydrator at 45°C for 8 h	*S*. Typhimurium (3); broth culture suspended in PS; applied and hand massaged	Room temp (undefined), vacuum packaged	56 days	2.5 log (drying); <1 log (storage)	Initial populations 7.5 log CFU/g. Soaking overnight at ambient temp led to population increases to 7–8 log CFU/g.	Kragh et al. (2022)
Mushrooms (enoki and wood ear), 2.5‐cm cubes	a_w_ 0.24–0.29	Dehydrated in laboratory at 60°C for 24 h before inoculation	Rifampin‐resistant serovars (4); broth culture washed in BPB; dried 24 h after inoculation	25°C, 33% RH	180 days	Enoki: ∼1.9, 2.9, and >4.3 log at 28, 90, and ≥120 days	Initial populations on both mushrooms were ∼6 log CFU/g. Enoki: 4‐log reduction (inactivation with a tail) at ∼140 days. Wood ear: ∼2 log decline during post‐inoculation drying.	Fay et al. (2023)
Peaches, halves	a_w_ 0.55	SO_2_ and non‐SO_2_ (total and free sulfur in treated fruit was 2,800 and 830 mg/kg, respectively)	Rifampin‐resistant serovars (5), and *E. faecium*; harvested from lawns suspended in PBS and added to bulk dried peaches and hand massaged	Room temp in zippered plastic bags	90 days	1.9 log for peaches treated with SO_2_ after 90 days; 3.3 log after 30 days on sulfur‐treated, and <LOD after 60 and 90 days	0.5–2.4 log reduction by acid pretreatments; *Salmonella* was detected in the pretreatment solutions; 60°C drying reduced populations by 1.5 log/peach; survived 90 days, with greater reduction observed on peaches made with SO_2_ treatment. *E. faecium* survived better than *Salmonella*.	Liu, Sheng, et al. (2022)
Pluots, dried halves	a_w_ 0.68, pH 3.4	SO_2_	Rifampin‐resistant serovars (5); agar lawn on TSA (rif); suspended in PBS	5, 20°C	180 days	>5.5 log at day 30 (5°C) or day 15 (20°C)	Initial population ∼8 log CFU/g after drying. LOD 1.9 log CFU/g. Not detected in 25‐g samples at day 30 at 5°C and day 15 at 20°C.	Canakapalli et al. (2022)
Raisins, whole	a_w_ 0.46, pH 4.08	None	Serovars (5); broth suspended in DW or PW (mist or dried on sand)	4, 25°C	242 days	Mist at 4°C: >2 log at 21 days Mist at 25°C: >5 log at 21 days	Initial populations 7.0 log CFU/g (mist) and 2.2 log CFU/g (sand). Detected in 25‐g samples 6/6: mist at 4°C for 242 days; (0/6): mist and sand at 25°C for 42 days; sand at 4°C for 42 days.	Beuchat and Mann (2014)
Saffron, whole stigmas	NS	None	Serovars (5); broth culture	20 ± 4°C	32 days	>6 log at 2 to 16 days	Strain and cultivar influenced rate of decline.	Pintado et al. (2011)
Strawberries, freeze dried slices	a_w_ 0.21, pH 3.32	None	Serovars (5); broth suspended in DW or PW (mist or dried on sand).	4, 25°C	242 days	Mist at 4 or 25°C: >2 or >3 log at 1 day	Initial populations 6.6 log CFU/g (mist) and 2.3 log CFU/g (sand). Not detected by enrichment (0/6): mist at 4 and 25°C, 242 and 84 days, respectively; sand at 4 and 25°C, 1 to 42 days (variable).	Beuchat and Mann (2014)
Tomatoes, sundried halves	a_w_ 0.78, pH 3.8	None	Rifampin‐resistant serovars (5); agar lawn on TSA (rif); suspended in PBS.	5, 20°C	180 days	>5 log at 60 days (5°C) and 15 days (20°C)	Initial 7–8 log CFU/g after drying. Not detected in 25‐g samples at day 15 at 20°C or day 60 at 5°C.	Canakapalli et al. (2022)
Tomatoes (Roma), halves	a_w_ 0.38–0.42, pH 4.0–4.5	Whole unblanched or blanched (88°C, 3 min), peeled and immersed in water, 3.4% ascorbic acid, or 0.21% citric acid (10 min)	Strains (5); broth culture, suspended in PBS	25°C	28 days	1.8‐2.6 log for control and water treated	Initial populations after drying at day 0 of storage were ∼4, 3.5, <1.3/1.5, and 2.2 log CFU/g for control, water, ascorbic and citric acid treatments, respectively (unblanched/blanched). After 28 days of storage, counts were 1.8/1.3, 1.4/1.7, <1.3, and <1.3 log CFU/g, respectively. Reductions on acid‐treated tomatoes during storage were ≤1 log because initial populations were at or below the LOD.	Yoon et al. (2004)
*Staphylococcus aureus*								
Spices, herbs	a_w_ 0.38 (parsley) to 0.47 (basil)	Steam, none	Strains (4), broth culture used to inoculate sand	Ambient (24 ± 2°C), dark	20 weeks	∼3 to >7 log at 10 weeks	Matrix effect observed for allspice, basil, cinnamon, nutmeg, oregano, paprika, parsley, pepper, with highest reduction in allspice and paprika, and lowest in cinnamon and parsley. D‐values ranged from 6.1 (allspice) to 16.3 days (cinnamon).	Dinh Thanh et al. (2018)

Abbreviations: BPB, Butterfield's phosphate buffer; BPW, buffered peptone water; DW, deionized water; LOD, limit of detection; NS, not specified; PBS, phosphate buffered saline; PS, peptone saline; PW, peptone water; RH, relative humidity; SO_2_, sulfur dioxide; SPB, sodium phosphate buffer; TSA, tryptic soy agar; TSAYE, tryptic soy agar with yeast extract; TSA‐YEP, tryptic soy agar with yeast extract and sodium pyruvate; TSB, tryptic soy broth

Reported differences in survival among pathogenic species during extended storage vary among studies, which may be attributed to storage conditions such as temperature or the method of inoculation. For example, populations of *E. coli* O157:H7 and *L. monocytogenes* declined below the limit of detection (>∼4 log reduction, none detected by enrichment) on dates (pH 5.4–5.8) after 90–120 days when stored at 20°C, compared to 150–180 days when stored at 5°C (Canakapalli et al. 2022). Under the same storage conditions, *Salmonella* spp. could be detected at ∼5 log CFU/g on dates stored at 5°C on Day 180, pointing to greater survival of *Salmonella* on low moisture dates at reduced temperatures compared to *E. coli* O157:H7 and *L. monocytogenes*. Similarly, *Salmonella* survival on dried mushrooms was reported to be better than that of *L. monocytogenes* (Kragh et al. 2022). STEC survived better in wet‐ than dry (sand)‐inoculated apricots compared to other pathogens (Liu et al. 2021). However, the survival of *Salmonella* was improved in sand‐inoculated apricots suggesting that differences among pathogens may be, among other variables, matrix‐ and inoculation‐carrier‐dependent (Liu et al. 2021).

Relative humidity can impact the water activity of dried products, which can subsequently influence the survival of pathogens during storage. Greater population declines were observed when dried spices were stored at higher relative humidity levels (Gradl et al. 2015; Ristori et al. 2007; Xie et al. 2022). In contrast, declines of *Salmonella* after 14 days of storage were greater on black pepper stored at 20% compared to 60% relative humidity (Li et al. 2020), suggesting that other factors likely influence survival. Nevertheless, when the water activity is not properly controlled during storage of dried matrices, surviving pathogens may be capable of regrowth when water activities become permissive. This might occur, for example, because of localized condensation. *Salmonella* grew in black pepper at high water activity (i.e., *a*_w_ of 0.98) (Keller et al. 2013), and during the rehydration of mushrooms in the home (Kragh et al. 2022).

Organic acids can be used as pretreatments to enhance microbial reductions during drying (Section 5). DiPersio et al. (2007) exposed cut carrots to blanching in citric acid, drying and subsequent storage, and demonstrated that citric acid treatment generally improved the reduction of *Salmonella* during drying and storage. A similar trend was observed for *Salmonella* in tomatoes pretreated with citric acid and ascorbic acid (Yoon et al. 2004), and for *L. monocytogenes* in peach slices dipped in ascorbic and citric acid prior to drying and subsequent storage (DiPersio et al. 2004). Pretreatment with sulfur dioxide has also been shown to enhance pathogen reduction during storage. The reduction of pathogenic *E. coli, L. monocytogenes*, and *Salmonella* during storage was higher on sundried apricots pretreated with sulfur dioxide (pH 3.5) compared to untreated controls (pH 4.3; Liu et al. 2021), coinciding with the lower pH of the sulfur dioxide‐treated apricots. Some products are naturally acidic with reduced pH levels, which can also affect pathogen survival. The survival of *E. coli* O157:H7, *L. monocytogenes*, and *Salmonella* was notably less on sundried tomatoes (pH 3.8) and dried pluots (pH 3.4–3.5), than on dates (pH 5.2–5.6) under the same storage conditions (Canakapalli et al. 2022).

## 7 Inactivation Treatments

In the past several decades, more research has been dedicated to evaluating current technologies and searching for alternatives that could be used to reduce pathogens on dried fruits, vegetables, herbs, and spices without significantly impacting sensory quality. However, these efforts face several challenges. When hydrated, intrinsic properties of the dried foods may prevent the comprehensive recovery of pathogens and confound the assessment of decontamination treatment efficacy. For example, many herbs and spices are known to contain essential oils that have antimicrobial properties (Chouhan et al. 2017), and fruits may have high acidity levels that make detection of microorganisms in (or on) them challenging, potentially leading to false negative results. To avoid these pitfalls, validated and verified laboratory methods for products investigated in the study should be used (e.g., ISO 16140 series) to achieve reliable results. In the following section, a detailed overview of the currently used and emerging inactivation technologies based on published studies is provided, outlining their advantages and potential drawbacks.

### 7.1 Thermal Inactivation Treatments

Thermal process controls used for pathogen reduction can be broadly categorized as moist heat treatments (e.g., steam or controlled humidity systems) and dry heat treatments (Table 5). The addition of moisture during treatment significantly reduces the time required to achieve equivalent pathogen reductions compared to dry heat technologies. However, relatively few studies have evaluated sensory attributes before and after treatment, limiting conclusions about the balance between microbial inactivation and product quality. In industry applications, thermal treatments are applied more frequently to dried vegetables, herbs, and spices than to dried fruits that typically do not undergo additional heat treatment prior to marketing. In one study, populations of pathogenic *E. coli*, *L. monocytogenes*, and *Salmonella* were reduced by >5 log on raisins and dried apricot halves with a temperature of 62–82°C during vacuum steam treatment (Acuff et al. 2020). However, the treatments slightly increased the water activity and corresponding moisture content to the extent that a subsequent drying step might be needed to ensure shelf stability.

**TABLE 5:** Thermal inactivation studies of foodborne pathogens on dried fruits, vegetables, herbs, and spices.

Treatment and matrix, form	Moisture (g/100 g) or water activity (a_w_)	Inoculation method	Inoculum (no. of strains)	Treatment conditions	Treat‐ment time	Treatment outcome	Comments	Reference
Aluminum test cells								
Dates, paste	a_w_ 0.45–0.46	Cell lawn; inoculated pre‐ and post‐fabrication into paste	*Salmonella* Enteritidis PT 30	80°C		*D_80°C_*: 3.5 min (pre), 1.2 min (post)	Differences in pre‐ vs post‐fabrication inoculation and equilibration influenced thermal resistance.	Limcharoenchat et al. (2018)
Black peppercorns	a_w_ 0.33, 0.54, 0.75	Cell lawn; peppercorns inoculated, then ground	*Salmonella* (5)	60–85°C	Up to 48 min	Predicted (extrapolated) 5‐log reduction at 75, 80, 85°C: 198, 40, 23 min at a_w_ 0.33; 272, 106, 43 at 70, 75, 80°C and a_w_ 0.54; 530, 29, 8 min at 60, 65, 70°C and a_w_ 0.75	*D* value increased with decreasing a_w_	Gautam et al. (2020)
Chili pepper; cinnamon; black pepper	a_w_ 0.3	Cell lawn	*Salmonella* (4)	70°C	Up to 2 h	*D_70°C_* at a_w_ 0.3: 15.4, 20.8, 36.6 min for chili, cinnamon, pepper, respectively	Better survival at lower a_w_, best in black pepper, worst in chili.	Xie et al. (2022)
Cinnamon, ground	a_w_ 0.20‐0.50	Cell lawn	*S*. Enteritidis PT 30	70, 75, 80°C	Up to 125 min	*D* values: 0.9–31.3 min, *z_T_* value: 11.9–18.2 C°, *z_aw_* value: 0.35–0.72	*D* value increased with decreasing a_w_.	Xie et al. (2021)
Dry heat								
Black peppercorns	ND	Broth and cell lawn	*S*. Typhimurium (3)	80°C	Up to 6 h	80°C, 180 min: 4.0‐log reduction (liquid); 2.3‐log (solid)	Solid inoculum ‐ higher resistance to heat.	Song (2022)
Black pepper, ground	a_w_ 0.40, 0.55, 0.70	Cell lawn	*Salmonella* (5); *Enterococcus faecium*	65–80°C	18 s to 250 min	*Salmonella* 𝛿‐value: 0.65–10.77 min, *z*‐value: 13.85–15.78 C°; *E. faecium* 𝛿‐value: 1.14–52.61 min, *z*‐value: 8.89–13.07 C°	𝛿‐value increased with decreasing a_w._ Non‐linear trend better described by Weibull model. *E. faecium* is suitable surrogate.	Wason, Verma, Wei, et al. (2022)
Black pepper, ground	a_w_ 0.25, 0.45, 0.65	Cell lawn; peppercorns inoculated and then ground	*Salmonella* (5); *E. faecium*	65–85°C	Up to 210 min	*Salmonella D* value: 2.0–46.0 min, *z* value: 11.9–13.0 C°; *E. faecium D* value: 1.5–67.6 min, *z* value: 11.8–13.7 C°	*E. faecium* is suitable surrogate.	Wei, Vasquez, et al. (2021)
Black peppercorns, whole and ground	a_w_ 0.40	Cell lawn	*Salmonella* (5); *E. faecium*	80°C	Up to 20 min	*Salmonella D _80°C_* 5.5 min pre‐grind, 3.9 min post‐grind	*E. faecium* is suitable surrogate.	Wason, Fuerte, et al. (2024)
Basil, ground	a_w_ 0.40, 0.55, 0.70	Cell lawn	*Salmonella* (5); *E. faecium*	70, 75, 80°C	Up to 80 min	*Salmonella D* value: 1.0–20.4 min, *z* value: 10.7–11.3 C°; *E. faecium D* value: 41.6–2.8 min, *z* value: 13.5–13.9 C°	*D* value increased with decreasing a_w._ *E. faecium* is suitable surrogate.	Verma, Chaves, Howell, et al. (2021)
Controlled RH								
Black peppercorns	a_w_ 0.43	Cell lawn, suspended cell pellet (sprayed)	*S*. Enteritidis PT30	80°C; 60, 70, and 80% RH	Up to 25 min	*D_80°C_* 5.1, 1.8, and 0.58 min at 60, 70, and 80% RH	Increased RH correlated with decreased thermal resistance.	Yang et al. (2022)
Steam								
Black peppercorns	a_w_ 0.35, 0.57, 0.69	Cell lawn	*Salmonella* (4); *E. faecium*	70, 75°C	5 min	Pathogens: ≥5‐log reduction at 75°C (smaller reduction at lower a_w_)	*Salmonella* most resistant among pathogens (*L. monocytogenes*, *E. coli* O157:H7); smaller reductions for *E. faecium* than for *Salmonella*. a_w_ increased during treatment.	Zhou et al. (2019)
Superheated steam								
Black peppercorns	12.2	Cell lawn	*Salmonella* (3)	100–180°C	Up to 25 s	*D_100°C_*: 4.6 s, *z*‐value: 47.1 C°	Greater increase in moisture at lower temperatures	Ban et al. (2018)
Garlic, cloves	ND	Spores	*Bacillus cereus*	120, 150, 180°C	Up to 3 min	≥5‐log reduction at 180°C with germinant pre‐treatment	Spraying of germinant compounds before treatment. Induction of spore germination increased reduction. Sublethal spore damage.	Jo et al. (2019)
Vacuum steam								
Raisins, whole	18.2‐25.0; a_w_ 0.56‐0.70	Cell lawn	*E. coli* O121:H19, O157:H7; *L. monocytogenes* (3); *Salmonella* (3); *Pediococcus acidilactici*	62 and 72°C under vacuum	Up to 20 min	Pathogens: *D_62°C_*: 3.0–3.1 min; *D_72°C_*: 0.7–0.8 min	*P. acidilactici* is suitable surrogate.	Acuff et al. (2020)
Apricots, halves, pieces	29.1‐31.8; a_w_ 0.68‐0.78	Cell lawn	*E. coli* O121:H19, O157:H7; *L. monocytogenes* (3); *Salmonella* (3); *P. acidilactici*	72 and 82°C under vacuum	Up to 20 min	Pathogens: *D_72°C_*: 2.3–2.5 min; *D_82°C_*: 0.6–0.7 min	*P. acidilactici* is suitable surrogate.	Acuff et al. (2020)
Black peppercorns	a_w_ 0.42	Cell lawn	*S*. Enteritidis PT 30	Pre‐heat 40°C, steam 75, 85, 95, 105°C	Up to 5 min	>5‐log reduction at all temperatures	Bacterial inactivation curves were non‐linear.	Shah et al. (2017)
Black peppercorns (BP); Cumin seeds (CS)	BP: 0.51, 0.33; CS: 0.38	Cell lawn, biofilm‐embedded cells	*Salmonella* (4); *E. faecium*	82°C	Up to 4 min	*Salmonella* BP: 5‐log reduction at 82°C for 83 s (cell lawn) or 202 s (biofilm); CS: 5‐log reduction at 82°C for 66 s	Higher resistance of biofilm‐embedded cells. *E. faecium* is suitable surrogate on BP, but not on CS.	Newkirk et al. (2018)

Abbreviations: ND, not determined; RH, relative humidity.

When evaluating thermal interventions for dried fruits, vegetables, herbs, and spices, factors influencing bacterial heat resistance in the specific product matrix need to be considered. For example, the state of the dried product at the time of inoculation or contamination may influence the efficacy of thermal treatments. For date paste, the *D*_80°C_ values were 3.5 and 1.2 min for pre‐ and post‐fabrication inoculation of the paste, respectively (Limcharoenchat et al. 2018). Although conducted with customized aluminum test discs in a water bath, the finding that pre‐fabrication contamination decreases the efficacy of thermal treatments may translate to larger scales. Similarly, when black pepper was inoculated prior to grinding (rather than after), elevated heat resistance of *Salmonella* was reported (Wason, Fuerte, Rojas, et al. 2024). In addition to the state of a low moisture matrix, microbial growth status may influence pathogen survival and heat resistance. Cells grown on agar plates (solid medium) exhibited greater heat resistance than cells grown in broth (liquid medium) (Song 2022). Similarly, increased heat resistance of *Salmonella* was reported when using a biofilm‐prepared inclusion method versus solid inoculum under the otherwise identical conditions (Newkirk et al. 2018).

Many published thermal inactivation studies on spices (Table 5) have focused on peppercorns or ground pepper, which are among the most widely used spice globally, and have been implicated in several outbreaks (Table 1). Most of these studies used moist heat. Ban et al. (2018) evaluated superheated steam treatments of peppercorns, and treatment temperatures of 100°C, 120°C, and 140°C increased moisture content to >15%, whereas treatments at 160°C and 180°C did not increase moisture, likely due to more rapid drying at the higher temperatures. These higher temperatures were also associated with higher inactivation rates, achieving >6 log reductions of *Salmonella* on peppercorns. Vacuum‐assisted steam treatments operating at temperatures <100°C have also been evaluated. Shah et al. (2017) reported >5 log reductions of *Salmonella* on peppercorns after 1 min at 75°C (lowest temperature applied) using a commercial vacuum steam unit in a pilot plant setting. Similarly, Newkirk et al. (2018) observed >5 log reductions after 1 min vacuum steam treatment at ≥83°C. A related study from the same group (Duncan et al. 2017) evaluated sensory changes following a 2‐min vacuum steam treatment and reported no visible differences but detectable odor changes, highlighting the need to assess sensory impacts for industrial applications. Cumin seeds treated in parallel with the black peppercorns did not exhibit sensory changes, underscoring the product‐dependent nature of thermal treatments. Zhou et al. (2019) evaluated steam treatment of black peppercorns in a chamber system and observed a 5‐log reduction of *Salmonella* after 5 min at 75°C across the water activities tested. Under the same conditions, pathogenic *E. coli* and *L. monocytogenes* strains demonstrated lower heat resistance than *Salmonella*, highlighting differences in heat resistance among strains and/or species. Steam treatment increases water activity in peppercorns, potentially requiring an additional post‐treatment drying step to maintain product stability during subsequent storage. Water activity significantly influenced heat resistance during dry heating. Gautam et al. (2020) and Wei, Vasquez, et al. (2021) adjusted the water activity of the whole peppercorn prior to dry heat treatments and observed decreased *D* values at higher water activity. Similar trends were reported in studies involving dried basil leaves (Verma, Chaves, Howell, et al. 2021) and spice powders (Xie et al. 2021; Wason, Verma, Wei, et al. 2022) where increased water activity corresponded to reduced heat resistance.

To address moisture and water activity increases associated with steam pasteurization, Yang et al. (2022) evaluated treatments using controlled high‐humidity air treatments rather than direct steam exposure. Condensation was prevented by preheating samples above the dew point temperature of chamber air. At 80°C, 60%, 70%, or 80% relative humidity achieved >5 log reductions of *Salmonella* after 25, 9, and 3 min, respectively, demonstrating that inactivation is more effective at higher relative humidities. Final moisture levels of the peppercorns were not elevated after treatment; at 60% relative humidity the moisture of the treated samples was lower than the untreated samples.

Variability in reported *D* values may also reflect differences among *Salmonella* serovars and strains. For example, different *D*_70°C_ values for *Salmonella* Enteritidis phage type 30 and a three‐strain cocktail were reported for cinnamon powder exposed to dry heat in two different studies (Xie et al. 2021, 2022) using similar methodology. Differences in *D*_70°C_ values may reflect strain‐specific thermal tolerance and should be considered when comparing studies.

Because many herbs and spices are harvested close to the soil or are derived from roots (e.g., garlic and ginger), they may be contaminated with high levels of spore‐forming bacteria, such as *B. cereus*. Jo et al. (2019) studied the effects of superheated steam together with germination‐inducing compounds (l‐alanine, inosine, and disodium 50‐inosinate) and reported that this approach significantly reduced the treatment time required to achieve 5‐log reductions of *B. cereus* compared to superheated steam alone.

### 7.2 Chemical Inactivation Treatments

Although chemical treatment of dried matrices may seem to be a good control measure to reduce bacterial loads, the approach should not be counterproductive, replacing one concern with another. The acceptance for use in foods and maximum residual level criteria of any chemical should be carefully assessed before considering the use of chemical agents. Although there are numerous references and options for chemical treatment of products from primary production and high moisture products, this option is poorly documented when directly applied on dried products. The persistence of the chemical compound and/or its derivatives in the treated product forms a definite hurdle to the implementation of such options in food processes.

A particular caution should be taken when it comes to ethylene oxide, a gaseous chemical treatment that has been used for decades to inactivate microorganisms in certain dried foods but is presently banned from use in most countries due to toxicological concerns (Dudkiewicz et al. 2022). Its use continues in some parts of the world, leading to major recall events in the EU, where more than 3,000 product recalls and notifications related to ethylene oxide have been reported since 2020. Although its use continues to be studied (Chen et al. 2021), it is no longer considered a viable treatment option for many commodities due to the regulatory limitations.

Gaseous chemical treatment for dried fruits, vegetables, herbs, and spices include acetic acid, chlorine dioxide, ozone, and ethylene oxide (with the abovementioned restrictions) (Table 6). Gaseous chlorine dioxide (ClO_2_), widely used and approved for other food types, has been assessed as a potential alternative to ethylene oxide processing in spices for control of *Salmonella* contamination. Although this technology is not currently approved by the US FDA for dried products, the expected lack of residues with ClO_2_ is an advantage over ethylene oxide. Studies have been conducted to assess the efficacy of ClO_2_ against *Salmonella* strains inoculated onto spices, with ClO_2_ generated either by passing a mixture of chlorine and nitrogen gas over a sodium chlorite cartridge (Wei, Verma, et al. 2021) or via a slow‐release sachet system consisting of gas permeable sachets containing a granular matrix impregnated with sodium chlorite precursor and a ferric chloride acid activator (Golden et al. 2019). A log‐linear inactivation of *Salmonella* was observed on black pepper and cumin seeds treated under various conditions of relative humidity, ClO_2_ concentration and hold times (Wei, Verma, et al. 2021), allowing a modified Bigelow model to be used to evaluate the effects of ClO_2_ concentration and relative humidity on *D* value. *Salmonella* populations declined by more than 5 logs in both products treated for 300 min at 15 mg/L ClO_2_ and 80% relative humidity. Ozone has also been considered for use on various matrices, with a limited lethal effect of ≤3 log at the highest concentration (Akbas and Özdemir 2008; Torlak et al. 2013). Apart from these gaseous treatments, other options considered for chemical treatment are advanced oxidation treatment (Görguç et al. 2021; Nasheri et al. 2021). The efficacy is nevertheless limited. The scarcity of technical solutions and the regulatory hurdles, not to mention consumer acceptance and the expectation of the food industry to aim for “clean labels” are elements explaining why the option of chemical inactivation is not commonly considered by food business operators.

**TABLE 6:** Chemical inactivation studies of foodborne pathogens on dried fruits, vegetables, herbs, and spices.

Treatment and matrix, form	Moisture (g/100 g) or water activity (a_w_)	Inoculation method	Inoculum (no. of strains)	Treatment conditions	Treatment time	Treatment outcome	Comments	Reference
Gaseous chlorine dioxide								
Black peppercorns (BP); cumin seeds (CS), sesame seeds (SS)	ND	Broth culture	*Salmonella* (5)	0, 100, 200, 500 mg/kg in gas permeable sachets, placed into drum mixers with samples	12 h	2.2–2.6 log reduction	Small increases in reduction at higher levels of chlorine dioxide.	Golden et al. (2019)
BP; CS	ND	Cell lawns, sprayed	*Salmonella* (5); *Enterococcus faecium*	5, 10, 15 mg/L, at 60, 70, 80% RH	60–300 min	1.5–5.4 log reduction after 300 min; >5 log in both spices at 15 mg/L, 80% RH, 300 min	Reduction increased with ClO_2_ conc. and RH% level. *E. faecium* is suitable surrogate.	Wei, Verma, et al. (2021)
Basil leaves (BL); BP; CS	ND	Cell lawns, sprayed	*Salmonella* (5); *E. faecium*	10 or 15 mg/L (BL); 5 or 10 mg/L (BP and CS), 15 mg/L, 80% RH	BL 125 or 150 min; BP 300 min; CS 300 min	*Salmonella*/ E. *faecium* reductions at higher mg/L and times: BL 3.6/4.1 log; BP 4.4/4.0 log; CS 4.0/2.8 log	Reduction increased with ClO_2_ conc. One week storage led to an extra ∼1 log decline for *Salmonella*. *E. faecium* is suitable surrogate.	Wei et al. (2023)
Gaseous acetic acid								
BP; fenugreek, seeds (FS)	10.5; 11.5	Broth culture	*Escherichia coli* O157:H7 (3); *Salmonella* Enteritidis (2)	0.3, 0.6, 4.7 mM gaseous acetic acid, 55°C	1, 2, 3 h	Log reductions after 3 h, 4.7 mM: *E. coli* >5.5 (BP), 5.6 (FS); *S*. Enteritidis >5.4 (BP), 4.8 (FS)		Nei et al. (2015)
Gaseous ethylene oxide (EtO)								
BP, CS	ND	Cell lawns, sprayed	*Salmonella* (5); *E. faecium*	735 mg/L EtO; 30, 40, 50% RH; 46, 56, 60°C	0–180 min	*Salmonella* 𝛿‐value ^a^: 0.02–3.6 min; *E. faecium* 𝛿‐value: 0.05–10.1 min (CS)	Reduction increased with increasing RH and temperature. Lower 𝛿‐values on BP. *E. faecium* is suitable surrogate	Chen et al. (2021)
Ozone								
Oregano, ground	ND	Broth culture sprayed onto product	*Salmonella* (3)	2.8 and 5.3 mg/L	30–120 min	Log reduction: 2.8, 3.7	Inactivation increased with O_3_ concentration.	Torlak et al. (2013)
Dried figs, whole	18.9‐19.3	Cell lawns sprinkled vegetative suspensions	*E. coli* (1); *B. cereus* (1) (cells)	0.1, 0.5, 1.0 ppm ozone at 20°C, 70% RH	360 min	Log reduction: *E. coli*, 0.9, 1.4, 3.5; *B. cereus*, 2.7, 2.9, 3.5		Akbaş and Özdemir (2008)
Dried figs, whole	18.9‐19.3	Spore suspensions, sprinkled	*B. cereus* (1) (spores)	1.0, 5.0, 7.0, 9.0 ppm ozone at 20°C, 70% RH	360 min	Log reduction: 1.0, 1.5, 2.0, 2.0		Akbaş and Özdemir (2008)
Combined treatments								
Figs, whole	21.0	Cell lawns, spot inoculation	*B. cereus* (1); *E. coli* O157:H7 (1)	Power‐ ultrasound (US) 527 W; PAA 148 mg/L	30 min	Log reduction of US and PAA: *B. cereus* 0.9; *E. coli* 3.4	US alone had least impact on *B. cereus*; in all cases <1 log.	Görguç et al. (2021)
Apple, raisins, strawberries	a_w_ 0.572 (raisins); 0.625 (apple); 0.699 (strawberry)	Broth culture centrifuged cell pellet sprayed onto product	*Listeria monocytogenes* (5); *Salmonella* (5)	PAA 1.25 to 5.0 parts per thousand (‰) using 20% v/v ethanol	2 h after treatment to ensure drying	Log reductions >4 *Salmonella* and *L. monocytogenes* for all products with 5‰	Increased effect with increased concentration.	Hasani et al. (2020)
Apple, raisins, strawberries	a_w_ 0.572 (raisins); 0.625 (apple); 0.699 (strawberry)	Broth culture centrifuged cell pellet sprayed onto product	*L. monocytogenes (5); Salmonella (5)*	UVC (54 mJ/cm^2^), ozone and/or H_2_O_2_ (10% v/v)	30 s in advanced oxidation process	Log reductions ≥5 *Salmonella*, >4 *L. monocytogenes* for all products with H_2_O_2_ alone and combined treatments		Hasani et al. (2020)
Dates, whole		Broth culture centrifuged cell pellet, spot inoculation	*E. coli* O157:H7 (4); *L. monocytogenes* (4); *Salmonella* (4)	75 ppm PAA alone; or + 70% EtOH; + 2.5% LA, + 70% EtOH + 2% trans‐cinnamaldehyde (TC)	1, 30, 60 s	4‐5 log reduction PAA alone (60 s) for all pathogens. Maximum log reductions >7 only reached for *E. coli* O157:H7 for PAA + 70% EtOH and PAA + 70% EtOH + 2% TC	No significant reductions on fresh whole dates compared to dried dates with those treatments.	Juneja et al. (2021)

Abbreviations: EtOH, ethanol; ND, not determined; LA, lactic acid; PAA, peracetic acid, RH relative, humidity. ^a^ 𝛿 is first decimal reduction.

### 7.3 Unconventional Inactivation Treatments

#### 7.3.1 Radio Frequency (RF) Heating

RF heating is an emerging thermal processing technology for food applications (Tong et al. 2022). During RF treatment dipole food molecules continuously align with the polarity of the alternating electric field and create molecular friction that is converted into thermal energy in the food. Compared to traditional thermal steam or hot air treatment, RF heating has a high heating rate due to high penetration capacity (Liu, Xiong, et al. 2022; Wason, Rojas, Subbiah 2024) and has a mild impact on food quality. During RF treatment, the food product is placed between two electrodes with a specified plate spacing; products may be treated in open containers or in sealed packages.

Pathogenic species of the Enterobacteriaceae family, that is, *Salmonella* and pathogenic *E. coli* (e.g., *E. coli* O157:H7) have most often been used to evaluate the decontamination efficacy of RF heating (Table 7). Some studies reported higher resistance of *Salmonella* compared to pathogenic *E. coli* (Tong et al. 2022), whereas others demonstrated similar reductions between the two pathogens (Jeong and Kang 2014; Kim et al. 2012). Up to a 6‐log reduction has been reported, depending on, among other factors, the treatment time and temperature profiles (Table 7).

**TABLE 7:** Inactivation studies of foodborne pathogens on dried fruits, vegetables, herbs, and spices using nonconventional process.

Technology and matrix, form	Moisture (g/100 g) or water activity (a_w_)	Inoculation method	Inoculum (no. strains)	Treatment conditions	Treat‐ment time	Log reduction	Comments	Reference
Cold plasma								
Figs, undefined	a_w_ 0.55‐0.52 after 12 min	Liquid inocula	*Escherichia coli* O157:H7 (1); *Enterococcus faecalis* (1)	High voltage DC pulse source with a maximum voltage of 17 kV and a current of 2.26 A. Gas flow rate was set to 10 standard L/min (sLm) and kept constant.	12 min	Max reduction after 12 min: *E. coli*: 2.6; *E. faecalis*: 2.1	Figs were pre‐dried using sodium metabisulfite and cabinet dryers.	Yarabbi et al. (2023)
Figs, whole	Initial a_w_ 0.70, 0.80, 0.93	Liquid suspension	*E. coli* O157:H7 (1); *L. monocytogenes* (1)	Microwave‐powered, 900 W, N_2_ gas 1 sLm (*E. coli*); 400 W, He‐O_2_ gas 1 sLm (*L. monocytogenes*)	10 min	*E. coli*: 0.5, 0.7, 1.3; *L. monocytogenes*: 1.0, 1.2, 1.6	Reduction impacted by a_w_.	Lee et al. (2015)
Black peppercorns; Onion, flakes	ND	Cell slurry	*S*. Enteritidis (1); *Enterococcus faecium* (1)	Indirect microwave‐powered plasma‐treated air (2.45 Ghz, 80W, 1.5 sLm)	1–30 min	1.5	Similar reduction of *Salmonella* and *E. faecium* on peppercorns; no reduction on onion flakes.	García Casado et al. (2024)
Peach, undefined	a_w_ 0.6 to 0.5 after 12 min	Liquid inocula	*E. coli* O157:H7; *E. faecalis*	High voltage DC pulse source with a maximum voltage of 17 kV and a current of 2.26 A. Gas flow rate was set to 10 sLm and kept constant	12 min	Max reduction after 12 min: *E. coli*: 3.0; *E. faecalis*: 2.3	Pre‐dried using sodium metabisulfite and cabinet dryers.	Yarabbi et al. (2023)
Black peppercorns	ND ^a^	Liquid suspension	*Salmonella* (1)	Direct radio frequency plasma jet with argon gas (12 mm distance to product, 10 sLm, 30 W); Indirect microwave‐driven plasma‐treated argon gas (2.45 Ghz, 1.2 kW, 22°C)	Direct 15 min; Indirect 30 min	Direct: 2.7 Indirect: 4.1	Higher inactivation observed with indirect than with direct. No effect on color quality. Slightly lower piperine concentrations.	Hertwig et al. (2015)
Black peppercorns	a_w_ 0.47	Cell lawn	*Salmonella* (4)	Electrode arc discharge plasma, 4 cm distance to product, 34 sLm (20 L air, 14 L argon)	80 s	∼5	Linear inactivation	Sun et al. (2014)
Black peppercorns	ND	Liquid suspension	*B. cereus* vegetative cells (1)	Direct plasma jet with air (9, 10, 11 cm distance to product (40, 50, 60 sLm; 800, 900, 1000 W); Indirect treatment in treated liquid	10 min	∼5 or more	Higher inactivation observed with direct than indirect. No effect on color, flavor, piperine, and phenol.	Wang et al. (2024)
Red (chili) pepper, ground	ND	Liquid suspension	*B. cereus* cells (1); *E. coli* O157:H7 (1)	Dielectric barrier discharge plasma (11.9 kV, 60 Hz, 10 mm distance between electrodes, argon 2.5 sLm)	15 min	*B. cereus*: <1; *E. coli*: ∼1	Further reduction (∼4 log *E. coli*) when treated powder was stored at 25°C.	Kim et al. (2022)
Cold plasma with pulsed light								
Red (chili) pepper, flakes	a_w_ 0.57	Liquid suspension followed with a_w_ re‐equilibration	*E. coli* O157:H7 (1)	Dielectric barrier discharge plasma generator (10 kV, 15 kHz), intense pulsed light generator (2.0 kV, 0.8‐3.8 Hz)	12.6 min	>3.8	No effect on color. Higher efficacy at higher a_w_ (0.73)	Lee et al. (2020)
Infrared heating with UV radiation								
Red (chili) pepper, ground	a_w_ 0.68	Liquid suspension	*S*. Typhimurium (3); *E. coli* O157:H7 (3)	Quartz halogen lamp, 350 mm, 500 W, 230 V	5 min	IR/IR with UV: *Salmonella*: 1.5/∼3); *E. coli*: 1.4/∼2.8		Ha and Kang (2013)
Irradiation (gamma)								
Cumin, seed; onion, ground; oregano, ground; black peppercorns	ND	Inoculated talc powder	*Salmonella* (2), *E. faecium*	24.5 Gy/min	0.5‐5 kGy	*D*‐values: *Salmonella*: 0.84–1.67 kGy; *E. faecium*: 1.10–1.70 kGy	*E. faecium* is conservative surrogate	Arias‐Rios et al. (2019)
Black and red (chili) pepper, ground	ND	Centrifuged cell pellets	*E. coli* O157:H7 (3); *S*. Typhimurium (3)	10 kGy/h	1–5 kGy	*E. coli* O157:H7: 4.3 to >5.2; *Salmonella* 3.8 to >5.1	*Salmonella* slightly more resistant than *E. coli* O157:H7	Song et al. (2014)
Irradiation (electron beam)								
Black peppercorns	ND	Cell lawn	*S*. Rissen (1); *E. faecium*	10 MeV high energy electron beam; 250 keV low energy electron beam	1–13 kGy	*Salmonella*: 5 at 4.2 kGy (HEEB), 8.1 kGy (LEEB)	*E. faecium* is conservative surrogate	Murdoch et al. (2022)
Black peppercorns	ND	Spot inoculation	Hepatitis A virus (HAV); murine norovirus (MNV)	10 MeV high energy EB	4, 8, 16 kGy	HAV: 2.1 at 8 kGy, >2.6 at 16 kGy; MNV: 0.3 at 16 kGy	Observable log reduction affected by low efficacy of recovery methods for viruses.	Butot et al. (2021)
Radio frequency (RF) heating								
Basil, leaves (in‐pack)	9.10–9.56	Cell lawns	*Salmonella* (5); *E. faecium*	16‐cm vertical (v) electrode gap; 10.5‐cm horizontal (h) electrode gap	115 s (v); 35 s (h	>6.5	Faster inactivation was observed in horizontal orientation. *E. faecium* is conservative surrogate. No significant quality loss.	Wason, Verma, Irmak, et al. (2022)
Basil, leaves (in‐pack)	9.26	Cell lawns	*Salmonella* (5)	27.12 MHz, 6 kW, 10.5‐cm electrode gap	40 s	>6	Use of steam vent retained the steam inside package, resulting in a relative low treatment time. No significant changes in color.	Wason, Rojas, et al. (2024)
Basil, leaves (in‐pack)	9.87	Cell lawns	*Salmonella* (5); *E. faecium*	27.12 MHz, 6 kW, 10.5‐cm electrode gap	65 s	>6.5	*E. faecium* is conservative surrogate. No significant quality loss.	Verma, Chaves, Irmak, et al. (2021)
Cumin, seed	<9.0	Cell lawns	*Salmonella* (5); *E. faecium*	27.12 MHz, 6 kW, 10.5‐cm electrode gap	100 s	*Salmonella*:>6.2; *E. faecium*: >6.5	*E. faecium* is suitable surrogate. No significant quality loss.	Chen et al. (2019)
Cumin, ground; paprika, ground; white pepper, ground	8.06; 11.42; 11.24	Cell lawns	*Salmonella* (4); *E. faecium*	27.12 MHz, 6 kW, 10.5‐cm electrode gap	125–375 s	*Salmonella*: 3.4–4.2); *E. faecium*: 1.4–1.9	*E. faecium* is conservative surrogate. Reduction in paprika > white pepper > cumin powder. No effect on color quality.	Ozturk et al. (2020)
Onion, ground	a_w_ 0.32	cell lawns, inoculated powder used to inoculate powder	*S*. Enteritidis PT 30	60, 65, and 70°C, 27.12 MHz, 8 kW, 80‐cm electrode gap; 51‐cm sample thickness	38 h	3.4	RF‐assisted heat treatment showed increased inactivation in comparison with traditional heat treatment.	Liu, Xiong, et al. (2022)
Black peppercorns (in‐pack)	10.7	Cell lawns	*Salmonella* (5)	27.12 MHz, 6 kW, 10.5‐cm electrode gap	115 and 155 s	2.7 to >6	Use of steam vent retained the steam inside the package for a while, resulting in lower treatment time. No significant changes in color.	Wason, Rojas, et al. (2024)
Black pepper, ground	ND	Centrifuged cell pellet	*E. coli* O157:H7; *S*. Typhimurium	27.12 MHz, 12.5‐cm electrode gap	7.0 and 8.0 min	>6	*Salmonella* was more resistant than O157. Model was developed for implementation of lethality model into RF process simulation. Color and flavor better retained compared with steam heating.	Tong et al. (2022)
Black pepper, ground	10.5	Sprayed cell suspension followed with a_w_ re‐equilibration	*Salmonella* (5); *E. faecium*	27.12 MHz, 6 kW, 10.5‐cm electrode gap	120 and 130 s	*Salmonella*: 4.0–5.9; *E. faecium*: 2.3–3.9	*E. faecium* is conservative surrogate. No significant quality loss. RF‐induced drying effect	Wei et al. (2019)
Black and red (chili) pepper, ground	10.1–30.5; 12.6–23.3	Centrifuged cell pellet	*E. coli* O157:H7; *S*. Typhimurium	27.12 MHz, 9 kW, 11‐cm electrode gap	80 s	>5	Reduction rate increased with moisture level to a maximum at 17.2% (black pepper) and 19.1% (red pepper) moisture. RF reduced moisture content and had a drying effect.	Jeong and Kang (2014)
Black and red (chili) pepper, ground; black peppercorns	ND	Cell suspension	*S*. Typhimurium; *E. coli* O157:H7	27.12 MHz, 9 kW, 11‐cm electrode gap	50 s (black pepper); 40 s (red pepper)	2.8–4.3 (black pepper); 3.4–3.5 (red pepper)	Slightly higher reduction rate with lower particle size. Similar survival for *Salmonella* and *E. coli* O157. No effect on color quality.	Kim et al. (2012)
Red chili pepper, ground (in‐pack)	4.4	Cell lawn	*S*. Enteritidis PT 30	27.12 MHz, 11‐cm electrode gap	20 min	5.8	No significant quality loss	Ma et al. (2022)
Red (chili) pepper, ground	11–21, a_w_ 0.57–0.74	Centrifuged cell pellet	*S*. Typhimurium (2)	27.12 MHz, 12 kV, 9.5‐cm electrode gap, 50–90°C	180 s	>5 (70°C, a_w_ 0.71, 170 s with come‐up time)	a_w_ affected inactivation, with highest reduction at 0.71.	Hu et al. (2018)
Ultraviolet light								
Bay leaves, whole	ND	Centrifuged cell pellet	*Salmonella* (7); *E. coli* O157:H7 (5); *L. monocytogenes* (2); *Staphylococcus aureus* (3)	UVC at 254 nm, 15 cm distance, 3,942 mJ/cm^2^	90 s	2.6–3.9	*E. coli* greatest reduction. Data followed non‐linear kinetics. Color and visible sensory properties not adversely affected.	Gabriel et al. (2020)
Garlic, ground; onion, ground; chili pepper, ground	a_w_ 0.36–0.43	Air dried pellet from broth culture	*E. coli* (1); *L. monocytogenes* (1); *S*. Typhimurium (1)	UVC‐LED at 270 nm for surface treatment, 2 cm distance, 16–128 mJ/cm^2^	40 s	0.75–3.0	Reduction in chili powder lowest (0.75–1.3 log). Data followed non‐linear kinetics.	Nyhan et al. (2021)
Black pepper, ground	11.9	Liquid suspension	*E. coli* O157:H7 (3); *S*. Typhimurium (3)	UVC (254 nm), 6 cm distance, 1.11 W/cm^2^	180 min	*E. coli*: 1.5; *Salmonella*: 0.9	Combined TiO_2_ coating increased UV inactivation. No effect on color quality. Moisture content decreased with 3.6%. Data followed non‐linear kinetics.	Park et al. (2020)
Red (chili) pepper, ground	ND	Liquid suspension	*E. coli* O157:H7 (3); *S*. Typhimurium (3)	UVC, 10 cm distance, 3.4 mW/cm^2^	10 min	*E. coli*: 0.5; *Salmonella*: 0.4	Combined with mild heat treatment (45–65°C) increased UV inactivation.	Cheon et al. (2015)

Abbreviations: IF, infrared; ND, not determined.

RF heating did not introduce significant changes in color or flavor substances (e.g., phenolics and/or volatile oil components) of spices and herbs (Chen et al. 2019; Kim et al. 2012; Liu, Xiong, et al. 2022; Ma et al. 2022; Ozturk et al. 2020; Tong et al. 2022; Verma, Chaves, Irmak, et al. 2021; Wei et al. 2019; Wason, Verma, Irmak, et al. 2022; Wason, Rojas, Subbiah, 2024) and retained these quality characteristics better when compared to steam heating due to the high‐temperature‐short‐time processing, which potentially minimizes the quality loss (Tong et al. 2022).

The moisture content is reduced during RF heating of unpacked products (Wei et al. 2019), and this may support optimal storage of the RF‐treated product (Wei et al. 2019) (Section 6). The moisture content (or water activity) may also affect the RF‐assisted inactivation efficacy. Faster inactivation has been reported in red pepper powder with higher moisture levels (Hu et al. 2018; Jeong and Kang 2014), in line with a higher rate of temperature increase (°C/s) at higher moisture content (Hu et al. 2018; Jeong and Kang 2014).

Nonuniform heating has been reported as a drawback of RF heating, including microwave heating technologies, and in‐pack treatment can improve the heating uniformity (Wason, Rojas, Subbiah, 2024). Steam venting packages can stimulate the circulation of the steam within a package and have been used for in‐pack RF treatment of dried basil leaves and black pepper (Wason, Rojas, Subbiah, 2024). These types of packages are also used in microwave‐treated products. In‐pack treatment could also be beneficial in preventing potential post‐processing recontamination (Wason, Rojas, Subbiah, 2024).

Kim et al. (2012) compared the survival of *Salmonella* and *E. coli* O157:H7 upon RF treatment of whole black pepper and of ground black peppers ground to various specifications with different mesh sizes. Faster inactivation rates were reported on the products with smaller particle sizes, corresponding to a higher temperature increase during treatment in ground black pepper compared with whole black peppercorns.

#### 7.3.2 Irradiation

Ionizing radiation technologies, such as gamma irradiation and electron‐beam (E‐beam) technology, use high‐energy rays to decontaminate dry products. These ionizing technologies are a non‐thermal alternative and cause disruption of the DNA and other macromolecules of microorganisms, rendering them inactive. E‐beam has more limited penetration depth than gamma irradiation, especially when low‐energy electron beams are used, because the delivered dose is concentrated at the surface of the target material (Ceylan et al. 2021; Murdoch et al. 2022). Reductions of ≥5 log could be achieved for *Salmonella* and *E. coli* O157:H7 on peppercorns irradiated at 5 kGy (Murdoch et al. 2022; Song et al. 2014), and maximum reduction (i.e., >2.6 log) of HAV was achieved on peppercorn irradiated at 16 kGy (Butot et al. 2021) (Table 7). Irradiation at high doses may adversely affect quality aspects of spices, as off flavors have been reported for red pepper powder (Jung et al. 2015).

#### 7.3.3 Ultraviolet Radiation

Ultraviolet (UV) radiation is a decontamination method commonly applied to liquids and surfaces. The UV spectrum is subdivided into UVA (315–400 nm), UVB (280–315 nm), and UVC (<280 nm); UVC is most frequently used because it represents the most germicidal wavelength range compared with the UVA and UVB (Dai et al. 2012). Some studies reported multi‐log reductions of microorganisms following UVC irradiation (Gabriel et al. 2020; Nyhan et al. 2021), whereas others observed no significant effect in dried spices (Cheon et al. 2015; Ha and Kang 2013) (Table 7). Studies that evaluated inactivation kinetics reported nonlinear survival curves with tailing (Gabriel et al. 2020; Nyhan et al. 2021; Park et al. 2020). UV irradiation can be combined with other mild treatments, such as mild thermal processing or infrared heating (Cheon et al. 2015; Ha and Kang 2013). Infrared heating, a surface‐based treatment, generally exhibits limited efficacy when applied alone (approximately 1–1.5 log), compared with conventional thermal treatment. However, when combined with other surface interventions such as UV irradiation, synergistic effects have been reported without measurable deterioration in product quality (Ha and Kang 2013).

#### 7.3.4 Cold Plasma

Plasma is an ionized gas and is considered the fourth state of matter, after solid, liquid, and gas states (Mandal et al. 2018). Non‐thermal plasmas are generated when high electrical energy is applied to a gas passed between electrodes, leading to ionization of the gas. The resulting plasma consists of reactive oxygen species, such as free radicals, electrons, ions, and different reactive chemical species depending on the carrier gas (Mandal et al. 2018). Argon, helium, nitrogen, or atmospheric air can be used as carrier gas, with the latter having the advantage that it is readily available. Non‐thermal plasma decontamination is a technology that is not yet available on the market for food processing (Ceylan et al. 2021). The decontamination efficacy is highly affected by the plasma source setup, and other technical, biological, and food parameters (Pampoukis et al. 2024). Plasma can be applied directly, or indirectly, when plasma‐processed air is being produced and subsequently used for surface product treatment. The generated reactive species can oxidize lipids in the cell membrane, leading to increased permeability or rupture, which disrupts the structural integrity of the cell and causes leakage of cytoplasmic content, ultimately resulting in cell death. Additionally, reactive oxygen and nitrogen species modify microbial proteins and enzymes, disrupting metabolic pathways and causing irreversible damage. High‐energy plasma species can also damage microbial DNA or RNA (Sasikumar et al. 2025).

Sun et al. (2014) reported a 5‐log reduction for *Salmonella* on black pepper, whereas others reported a significantly lower reduction (García Casado et al. 2024; Hertwig et al. 2015) (Table 7). The differences in decontamination efficiencies among studies indicate the potential for optimization of plasma treatment of dry matrices, and/or in combination with treatments using other mild technologies or extended storage of the plasma‐treated matrices (see, e.g., García Casado et al. 2024; Kim et al. 2022; Lee et al. 2020; Sun et al. 2014; Wang et al. 2024). A reduction of 2.6 log was achieved for *E. coli* O157:H7 in dried figs, whereas reductions on dried peaches were higher, at 3.0 log, with no significant effect on sensory characteristics (Yarabbi et al. 2023).

Another unconventional variation of cold plasma is microwave‐powered plasma treatment. Although microwave plasma treatment is mostly non‐thermal, localized heating can occur, contributing to microbial inactivation in some cases. Lee et al. (2015) showed that microwave‐powered plasma treatment resulted in 1.3 and 1.6 log reductions of *E. coli* O157:H7 and *L. monocytogenes*, respectively, on dry figs at water activities of 0.91 and 0.90, respectively. Smaller reductions were observed at reduced water activities for both microorganisms.

#### 7.3.5 Use of Surrogates to Validate Inactivation Treatment

For commercial food processors, preventive controls used to manage biological hazards must be validated to demonstrate that they can effectively control the target pathogen(s) and comply with applicable regulatory requirements (Ceylan et al. 2021). Although process validation can be conducted using several approaches, the use of surrogate microorganisms with characteristics, similar to those of the target pathogen, is a robust and widely accepted strategy (Hildebrandt and Marks 2024; Hu and Gurtler 2017). Data should be generated to demonstrate that the selected surrogate is at least as resistant as, or more resistant than, the target pathogen in the selected matrix and under the intended treatment conditions.

*E. faecium* ATCC 8459 (also designated NRRL B‐2354; Kopit et al. 2014) has been used as a surrogate in several thermal inactivation studies evaluating treatments to control *Salmonella* in dried fruits, vegetables, herbs, and spices, demonstrating suitability in many, but not all, applications. This organism was more resistant than *Salmonella* during RF heat treatment of a range of herbs and spices (Ozturk et al. 2020; Verma, Chaves, Irmak, et al. 2021; Wason, Verma, Wei, et al. 2022; Wei et al. 2019)*. E. faecium* exhibited greater heat resistance than *Salmonella* on black peppercorns treated with vacuum steam, whereas reductions on cumin seeds were not significantly different and the authors concluded that *E. faecium* was not a conservative surrogate under those conditions (Newkirk et al. 2018).

Several studies also demonstrated that *E. faecium* NRRL B‐2354 is a suitable surrogate for *Salmonella* under unconventional inactivation treatments for several herbs and spices. For example, *E. faecium* NRRL B‐2354 showed promise as a conservative surrogate for *Salmonella* during gaseous ClO_2_ treatment of spices although it was less appropriate as a surrogate during post‐treatment storage (Wei, Verma, et al. 2021; Wei et al. 2023). This organism was also more resistant than *Salmonella* during gamma and E‐beam irradiation of spices (Arias‐Rios et al. 2019; Murdoch et al. 2022), whereas it showed similar microbial reductions for plasma‐processed air treatment of black pepper (García Casado et al. 2024). *E. faecium* ATCC 33186 has been evaluated as a surrogate strain for *E. coli* O157:H7 for cold‐plasma‐treated dried peaches and figs (Yarabbi et al. 2023), where it appeared to be a conservative surrogate. Collectively, these studies emphasize the importance of confirming the suitability of surrogate organisms for the specific target matrix and treatment technology.

## 8 A Simplified, Qualitative Risk‐Based Framework for Industry Decision‐Making

Dried fruits, vegetables, herbs, and spices are used in many ways in complex food supply chains. They are sold directly or via brokers to retail consumers or to food processing or food service customers that incorporate these ingredients into their products. Microbiological risk assessment, management, and communication at all stages of the supply chain are important to evaluate if a dried ingredient can be safely consumed without additional risk‐reduction step(s) by the customer (business‐to‐business) or consumer. In Tables 8 and 9, a framework for guiding these assessments and facilitating communication is proposed, applying the information collected in the present review.

**TABLE 8:** A tool for applying a risk‐based decision framework for microbiological safety of dried fruits, vegetables, herbs, and spices.

Topic	Question	Answer	Risk Score
Pathogen prevalence indicators			
(1) Historical evidence: Recalls, outbreaks, regulatory notifications, or prevalence data	Has a recall or outbreak been attributed to the material type or similar material type, or is there an indication from regulatory import notification or the scientific literature that a pathogen is prevalent in the material?	No recalls, outbreaks, or notifications. Multiple sources reporting no pathogen detection. Recall(s), notification(s), or scientific literature indicating pathogens found in the material type. No associated outbreaks. Outbreaks(s)	1 5 15
Contamination potential BEFORE or DURING pathogen reduction step(s)			
(2) Agricultural system contamination risk	Are raw materials sourced from farms using Good Agricultural Practices (GAPs)?	Yes No	1 5
(3) Crop exposure to soil and irrigation water	Is the crop excluded from direct contact with soil and/or irrigation water?	Yes No	1 5
(4) Pre‐drying storage, handling, and processing contamination risk	Do pre‐drying storage, handling, and processing conditions prevent pathogen growth and preclude potential contamination during the process (e.g. fluming, peeling, slicing, fermentation, water soaking)?	Yes or not applicable No	1 5
(5) Location of drying process	Does the drying process occur in a controlled, indoor facility that is routinely cleaned and sanitized (e.g. in an oven inside a manufacturing facility vs. sun‐dried outdoors)?	Yes No	1 5
Pathogen reduction step(s)			
(6A) Pre‐drying treatment	Is there a pre‐drying treatment that is validated to reduce pathogen levels (e.g. blanching, sulfite, ascorbic acid)?^a^	≥ 5 log CFU/g reduction 2.5‐5 log CFU/g reduction No or < 2.5 log CFU/g reduction	1^b^ 25^c^, ^d^ 50^e^
(6B) Drying	Are the drying conditions alone validated to reduce pathogen levels?^a^	≥ 5 log CFU/g reduction 2.5‐5 log CFU/g reduction No or < 2.5 log CFU/g reduction	1^b^ 25^c^, ^d^ 50^e^
(6C) Post‐drying treatments	Is there a post‐drying treatment that is validated to reduce pathogen levels (e.g. steam, irradiation, ozone)?^a^	≥ 5 log CFU/g reduction 2.5‐5 log CFU/g reduction No or < 2.5 log CFU/g reduction	1^b^ 25^c^, ^d^ 50^e^
(6D) Storage of dry ingredient	Are storage conditions of the dry ingredient prior to shipment validated to reduce pathogen levels?^a^	≥ 5 log CFU/g reduction 2.5‐5 log CFU/g reduction No or < 2.5 log CFU/g reduction	1^b^ 25^c^, ^d^ 50^e^
Contamination potential AFTER pathogen reduction step(s)			
(7) Cross‐contamination control	How effective are pathogen cross‐contamination control programs after the pathogen reduction step(s) (if applicable)?	Effective. Pathogen cross contamination is unlikely. Marginally effective. Programs have gaps that might result in pathogen cross contamination. Ineffective. Programs are absent or have major gaps that make pathogen cross contamination likely. Or, not applicable because there is no pathogen reduction step (question 6 score is 50).	1 15 30
Overall Risk Score (sum of 1‐7)^f^			7–115

*Note*: An interactive version of this table is available in Table S1.

^a^ Use the minimum log CFU/g reduction that was validated to answer this question. Do not use the average log CFU/g reduction.

^b^ If A, B, C, OR D = 1, then (6) = 1, regardless of other scores.

^c^ If one of A, B, C, OR D = 25 and none = 1, then (6) = 25.

^d^ If ≥2 of A, B, C, OR D = 25, then (6) = 1.

^e^ If A, B, C, AND D = 50, then (6) = 50.

^f^ Recommendations based on overall risk score are in Table 9.

**TABLE 9:** Recommendations based on overall risk score from Table 8.

Overall risk score	Recommendation
≤30	Pathogen risk‐reduction measures in the ingredient supply chain are considered adequate to ensure product safety. No additional risk‐reduction step(s) by the customer or consumer are required
31–55	Additional actions to further reduce pathogen risk are recommended. They may include the following: Implementing additional pathogen risk‐reduction measures within the ingredient supply chain, and/or Incorporating risk‐reduction measures by the end user (customer or consumer). If additional risk‐reduction measures are not permitted or feasible, a comprehensive risk assessment can be conducted. This assessment should consider factors such as raw material origin, supplier performance, historical data, pathogen prevalence, and typical amount consumed in the specific application. If the outcome is deemed acceptable by company leadership and subject‐matter experts, the ingredient may be used provided that all evaluated controls and assumptions are maintained
≥56	Ingredient is not recommended for use without additional pathogen‐reduction step(s) implemented by the customer or consumer

The output score of the risk‐based framework tool is weighted to favor steps that impact microbiological safety. Historical recalls, outbreaks, and regulatory notifications can help inform the likelihood of contamination occurrence; however, the absence of historical events does not suggest that contamination cannot occur. Likewise, pre‐lethality‐step handling is important to reduce contamination likelihood and to prevent a significant increase in a contaminant, but it cannot significantly reduce pathogens that may have been introduced during field production. Validated and controlled pathogen reduction and cross‐contamination prevention measures are more heavily weighted because these steps provide proven, reliable pathogen control when managed properly.

The risk‐based framework is intended to facilitate the practical application of the extensive information reviewed in this paper by providing simple, qualitative, and directional guidance to commercial vendors and buyers of these dried materials so that safe applications are selected for the end consumers. The framework is proposed as a simplified guideline, acknowledging that there are many factors and nuances that impact the microbiological safety of dried fruits, vegetables, herbs, and spices that may affect each topic. The framework scoring system is intentionally weighted so that the strongest indicators of microbiological risk and the most effective risk reduction actions have the biggest effect on the overall risk score. The scoring system was developed through expert consultation, extensive testing of hypothetical and real scenarios, and iteration. The overall risk score provides a starting point for procurement discussions, additional information gathering, and more detailed and complex risk assessments, as needed.

Some of the topics in the framework may become part of a contracted specification between companies or criteria used for vendor selection for a one‐time purchase (e.g., country of origin, on‐farm practices, and pathogen reduction step). Therefore, the breadth of material to which the framework applies depends on the specific use case (e.g., one batch vs. every batch purchased under a contract). The breadth is determined by the user of the framework. For longer term arrangements, the framework should be revisited when there is new information or a change to the specification.

The discussion below includes further explanation of the weighted score system, and considerations and limitations for each of seven topics that are addressed in the risk‐based framework.

### 8.1 Recalls, Outbreaks, Regulatory Notifications, or Prevalence Data

Historical evidence of pathogen contamination, including outbreaks, recalls, and regulatory notifications, can provide an indication of the likelihood and/or prevalence of pathogens in a dried ingredient (Sections 2 and 3). Internal industry data generated through incoming ingredient testing and supplier verification programs may also be useful. Published scientific literature may also provide insights into pathogen prevalence (Section 3). No fixed timeframe is specified for evaluating these data sources as relevance may depend on factors such as the ingredient type, country of origin, changes in production or processing practices, supplier history, and the availability of current information. When evaluating available data, consideration should be given to the specific raw material, country of origin, handling and processing details, supplier, and root cause of contamination, if available, in determining the relevance of the data to the material being assessed. Data from similar materials should also be reviewed. Similarity of materials may be based on raw material form (e.g., root, seed, leaf, and fruit), pH, processing steps (e.g., peeling or no peeling, whole or sliced), and other relevant characteristics. For example, results for peeled, dried apple slices should be considered when evaluating peeled, dried peaches, although results for dried carrots would be less relevant to peaches given the differences in raw material form and pH. Importantly, the absence of available data does not indicate the absence of pathogens in an ingredient type; rather, it may reflect limited surveillance, inadequate sampling or under reporting. On the other hand, multiple studies reporting no pathogen detection in a specific ingredient type may be more indicative of low prevalence. In the scoring framework, historical evidence has a moderate effect on the overall risk score, with a score range of 1–15. This topic is weighted moderately because many factors can influence whether pathogens are detected in dried materials as reported by regulatory agencies, scientific research, and the food industry. Factors affecting data availability and test results include the following: the material's processing history prior to sampling, any intended downstream processing, potential bias in research funding and regulatory sampling, and differing test methodologies and regional monitoring frequencies. Answers are weighted most heavily for materials associated with a known foodborne disease outbreak(s), as such outbreaks suggest that pathogen levels were higher than expected or that control measures were insufficient or improperly applied. This may indicate an incomplete understanding of contamination routes and levels within the supply chain or inadequate controls and therefore justifies a higher score.

### 8.2 Agricultural System Contamination Risk

GAPs are principles, guidelines, and procedures that help reduce the potential for pre‐ and postharvest pathogen contamination of produce, among other objectives (Section 4). Guidelines address employee hygiene, water quality, site location, pest management, and other factors that may contribute to pathogen access to the crop. Growers may follow the principles with or without having a formal GAP certification verified by periodic audits. However, when evaluating supply chains, a formal certification provides a third‐party verification of adherence to the guidelines.

### 8.3 Crop Exposure to Soil and Irrigation Water

Because soil and water can harbor and transfer pathogens to produce (Gurtler and Gibson 2022; Jongman and Korsten 2017; Uyttendaele et al. 2015), controlled environment agriculture methods, such as hydroponics and aeroponics, that do not use traditional soil and irrigation water may provide a lower risk of pathogen contamination when managed properly (Ivey et al. 2025). Because most crops are not produced this way, it is recommended to answer “no” to this question unless there is specific knowledge of specialty growing conditions.

### 8.4 Pre‐Drying Storage, Handling, and Processing Contamination Risk

Before the material is dried, several processing steps may occur in which there is potential for pathogen growth and/or contamination. If wash and flume water is not maintained properly, it can become a source of contamination. Sanitizer added to wash and flume water can prevent or minimize pathogen growth and cross‐contamination (López‐Gálvez et al. 2021). Water alone or water containing sanitizer can reduce pathogens on produce by ∼0.5–2 log; however, in practice, conditions to ensure a consistent reduction can be difficult to control (Beuchat 1998, Gonzalez et al. 2004, López‐Gálvez et al. 2019). Therefore, the potential pathogen reduction with washing and fluming is not considered in this framework. If a validated and controlled wash process exists, it could be considered under topic 6A in the framework. Similarly, if peeling and cutting equipment are not properly maintained, cleaned, and sanitized, they can become a source of pathogens. Processes involving water soaking or fermentation prior to drying must also be managed carefully to ensure that pathogens are not introduced and do not have an opportunity to multiply.

### 8.5 Location of Drying Process

Sun‐drying is common for some dried ingredient types such as black peppercorns and raisins. Materials dried outside have a higher potential contamination risk from the environment and animals when compared to those dried in a controlled indoor facility with Good Manufacturing Practices, pest control, and sanitation practices. Sun drying is slower than oven drying that exposes the material to the environment for a longer time, and fans used to speed sun‐drying may distribute contaminated material in the environment. Scientific studies on the relative risk of outdoor and indoor food drying are lacking; therefore, considering the risks associated with specific outdoor drying operations is recommended.

Framework Topics 2–5 cover the risk of pathogen contamination or proliferation during growing, harvesting, pre‐drying storage, handling, and processing. Collectively, these topics have a moderate impact on the overall risk score, contributing 4–20 points. Although strong management of these steps is important for minimizing pathogen spread and growth, vendors and buyers of dried ingredients may be several steps away from these operations. In addition, raw materials may be co‐mingled from multiple farms or geographic regions. Consequently, buyers may have limited ability to evaluate these controls or to rely on them as validated steps for pathogen lethality or removal.

### 8.6 Pathogen Reduction Steps: Drying, Storage, and Treatment (Sections 5, 6, 7)

Although framework Topics 1–5 addressed reducing the risk of contaminating a raw material with a pathogen, Topic 6 addresses steps that can significantly reduce pathogens on contaminated raw material. Because these steps actively reduce pathogen levels, they are weighed more heavily than the previous steps. Reduction of pathogens is strongly impacted by the raw material, the specific pre‐ and post‐drying treatments, as well as the drying process. Pre‐drying treatments may be used to preserve the color and structure of some dried ingredients. These treatments may also reduce pathogen levels under certain conditions (Table 3). When these treatments are validated with a documented pathogen reduction level and controlled, they can be considered part of a pathogen control program. In addition, the drying step itself can sometimes serve as a pathogen reduction step depending on the drying conditions and starting material. A post‐drying inactivation treatment may be applied to the dried ingredient specifically to reduce pathogens. Various chemical and physical technologies are employed for this purpose (Section 7, Tables 5, 6, 7). When using pre‐drying, drying, or post‐drying steps as pathogen reduction control step(s), it is critical that the parameters used during processing are the same as or more severe than those used during process validation (e.g., higher temperature or longer time than the validated conditions). Other considerations are regulatory permissibility of the treatment and the impact of the treatment on sensory attributes.

Lastly, storage of the dried ingredient over time can cause significant reduction in pathogen numbers depending on the specific ingredient's attributes and storage conditions (Section 6, Table 4). Storage time is not commonly included as part of a pathogen control program due to the high cost of extended temperature‐ and humidity‐controlled storage conditions. However, if carefully validated and controlled, storage time and conditions could be considered a lethal step when shown to result in significant and consistent reduction of pathogens in the specific ingredient.

All pathogen reduction steps included in the pathogen control program should be validated and controlled to ensure that minimum validated conditions are met. For simplicity, specific pathogens are not mentioned in the following discussion, but in general vegetative bacterial and viral pathogens are the primary concerns in low water activity foods. Spores do not pose a risk in the dried material itself because the low water activity prevents germination and growth required for spores to pose a risk. However, spores should be considered in the overall finished consumer product design if the product formulation or intended use supports spore germination and growth. This consideration was outside the scope of the present risk assessment framework for dried ingredients. Validation studies should be reviewed by an expert to ensure correct experimental design and data interpretation. Common pitfalls in lethality validation studies include using the incorrect target pathogen or surrogate organism (i.e., failing to use the most treatment‐resistant pathogen likely to occur or an appropriate surrogate); failing to base the lethality validation on a pathogen or its surrogate (e.g., using aerobic plate count, coliform counts, or yeast and mold counts); failing to adapt the pathogen or surrogate to the conditions of the test material (e.g., not drying the inoculum to best represent the natural state of the organism in a dried material); failing to validate the worst‐case product and processing conditions; and using the average, rather than  the minimum, log reduction. More information on pathogen lethality validation methods, design, and interpretation can be found in scientific literature (Anderson and Lucore 2020; Ceylan et al. 2021; Wason et al. 2021).

The framework considers a 5‐log reduction of the most resistant pathogen or surrogate in the matrix, or a comparable matrix as sufficient to ensure food safety or to represent a “full lethality” treatment (Schaffner et al. 2013). Schaffner et al. (2013) further suggest that this target is appropriate for low‐moisture foods when sufficient data for a quantitative microbial risk assessment (QMRA) are not available, provided that initial contamination levels of vegetative pathogens are low (<1 CFU/g), and the process is well controlled. However, commodity‐specific risk assessments or regulatory frameworks may establish alternative lethality targets depending on product characteristics, prevalence and concentration data, and anticipated consumption patterns. For example, a minimum 4‐log reduction of *Salmonella* in almonds grown in California and sold in North America is required under the Almond Board of California (Federal Register 2007) based, in part, on several QMRAs (Danyluk et al. 2006; Lambertini et al. 2012; Santillana Farakos et al. 2017b). Similarly, US FDA QMRAs for other tree nuts have identified target reductions for *Salmonella* ranging from 3 to 4 log depending on the commodity and associated risk profile (Santillana Farakos et al. 2017a, 2017b, 2018, 2019). Comparable detailed QMRAs are not currently available for dried herbs, spices, fruits, and vegetables. However, the principles within the framework can be adapted to different lethality requirements by adjusting the target log reductions in topics 6A to 6D.

The framework allows two or more partial lethality steps (2.5–5 log reduction) to be combined and give the same score as a full lethality step (≥5 log reduction). This additive approach is only valid if the starting material for each process step is consistent with the starting material in the relevant validation study. For example, if a dried, chopped onion is processed by chopping, drying, and then steam treating, it is reasonable to add the log reduction for the drying step and the log reduction for the steam treatment if the steam treatment validation was done on dried, chopped onions. If the steam treatment validation was completed on fresh chopped onions, it cannot be added to the drying lethality for the process. Ceylan et al. (2021) provide additional considerations for a series of process steps.

The presence or absence of a pathogen reduction step(s) is the most heavily weighted of the seven scores in the framework (up to 50 points) and has a high impact on the overall risk score. A validated pathogen lethality step allows the dried ingredient manufacturer more control over the pathogen risk and allows the dried ingredient buyer to evaluate the process and include it in ingredient specifications and contracts. This step is more heavily weighted than previous topics because a well‐designed and validated pathogen reduction step(s) can overwhelm typical early supply‐chain pathogen contamination levels.

### 8.7 Cross‐Contamination Control

Pathogen cross‐contamination from equipment, the environment, and raw materials that have not undergone a pathogen reduction step must be controlled after a pathogen reduction step is applied to ensure that the ingredient is not recontaminated. Pathogen cross‐contamination control measures include Good Manufacturing Practices, hygienic zoning, equipment and infrastructure maintenance, and effective equipment, environment, and transportation sanitation procedures (Karappuchamy et al. 2024; Lucore et al. 2017). An effective environmental monitoring program will help verify efficacy of cross‐contamination control measures and identify potential issues for corrective action before product contamination can occur (Bourdichon et al. 2021).

Failure to control cross‐contamination negates or reduces the impact of the pathogen‐reduction step; thus this topic is weighted heavily in the final risk score. When post‐lethality step pathogen cross‐contamination control programs are absent or ineffective, the score is 30. An example of pathogen cross‐contamination control absence is when treated materials are run on the same system as untreated materials without a validated sanitation in between. Ineffective programs are revealed via on‐site audits, procedure reviews, and monitoring results. Examples include poor facility maintenance with unsanitary leaks dripping on product zone, sanitation SOP with ineffective sanitizer concentration, or product/environment monitoring results indicating high likelihood of pathogens contaminating the product or product contact zone. Marginally effective programs receive a score of 15 and may include persistent facility maintenance issues in the manufacturing area that do not impact product zone, gaps in sanitation procedures or execution, or monitoring results that indicate a potential for pathogen contamination of the product or product contact zone. The lowest score (1) is given when cross‐contamination programs are well‐developed and executing such that there is a low likelihood of pathogen contamination.

When using the risk‐based decision framework, assuming the worst case scenario in the absence of information is recommended. The framework may overestimate risk in these cases but will also highlight data gaps for future research and data collection. In addition, when the source of data is not clear or there is low confidence in the data quality or data relevance to the process or ingredient, a conservative approach is encouraged.

Importantly, testing the dried ingredient for a pathogen is not a contamination control or a pathogen reduction step. Simply testing the material for a pathogen and not detecting it does not mean that the entire tested ingredient lot is free from the pathogen or that a control step has been effective (Jongenburger et al. 2015). Pathogen testing is one method of verifying a pathogen control but should be used in conjunction with other verification activities. Typical natural pathogen contamination levels and practical sampling levels are too low for pathogen test results to be used to determine that a material was not contaminated or that a process or treatment was effective. Proactive, validated, and verified programs and processes are the ways to routinely ensure the safety of these ingredients. Routine pathogen testing can be useful to detect unusually high contamination events in materials. Significantly increasing the sampling level (number and/or size of samples) for a lot for investigation purposes increases the sensitivity of pathogen testing exercise but is not practical or economical for routine use.

Table 9 provides recommendations on how to react to the risk score from the framework in Table 8. Scores of ≤30 indicate that risk reduction measures are considered adequate to ensure safety without additional risk reduction step(s) by the customer or consumer. These ingredients have a minimum of a validated pathogen reduction step with cross‐contamination control measures or a lower likelihood of initial contamination. Ingredients with scores between 31 and 55 need further assessment to determine the appropriate course of action. Where possible, ingredients with a score of 31–55 should be replaced with an ingredient with a score of ≤30 or the ingredient should be directed toward applications where the customer or consumer will further process to provide a pathogen reduction. When neither of the above solutions are permitted or feasible, a risk assessment should be documented by a microbiological food safety expert and the critical food safety drivers documented and maintained. Alignment with appropriate company leaders is also recommended to ensure organizational risk communication and to gain support for future risk reduction. Ingredients with scores of ≥56 are not recommended for use without further processing by the customer or consumer to reduce pathogens. Disclosure of the safe intended use of the ingredient to customers and/or consumers promotes awareness that they must cook or otherwise process the ingredient for pathogen reduction. Package statements such as “Not intended for consumption without further processing to reduce pathogens” for a food manufacturing or food service customer or “Cook thoroughly before consumption for safety” for a retail consumer are options for disclosure. Common consumer misuses of retail foods should be considered in determining if it is appropriate to use an ingredient with a score of ≥56 in products relying on a consumer pathogen reduction process.

An interactive version of the framework in Table 8, including the recommendations in Table 9, is available in Table S1. Also included are hypothetical examples of dried fruits, vegetables, herbs, and spices with different risk scenarios. The answers to the questions in the examples are truly hypothetical and should not be interpreted as guidance for answering any question. For example, a freeze‐dried berry where lethality has not been determined would be in the highest risk category with a score of 97 (a score of 50 for topic 6 is the primary driver of the high score), whereas a similar berry that is dried at low temperatures (3‐log reduction) or dried at high temperatures (>5 log reduction) would have overall scores of 43 and 19, respectively (topic 6 scores of 25 and 1), putting them in the medium or low risk categories, respectively. The interactive tool allows users to evaluate different ways to reduce the risk in the supply chain.

The scoring approach and recommendations are intended to guide suppliers and customers of dried fruits, vegetables, herbs, and spices in sourcing, process development, and microbiological risk assessment so that safe foods are available to consumers and for regulatory compliance. The main factors affecting microbiological risk of these ingredients are included in the framework; however, other factors specific to a particular material, supplier, or region may also affect microbiological risk and should be included in the overall risk determination with guidance from a microbiologist.

## 9 Conclusions

Dried fruits, vegetables, herbs, and spices are globally traded commodities valued for their long shelf life and diversity of applications in foods, often without a subsequent microbial reduction step (e.g., cooking) prior to consumption. Accordingly, the microbiological safety of these commodities has been evaluated in this review. Outbreak and recall data, together with prevalence studies, demonstrate that a number of bacterial pathogens and viruses are food safety concerns. However, standardized prevalence studies are lacking, and available data may be influenced by targeted investigations conducted in response to outbreaks. Nevertheless, microbial contamination remains a concern in dried fruits, vegetables, herbs, and spices and must be managed throughout the supply chain. Effective management begins with application of GAPs during primary production and harvest to minimize microbial contamination and support the efficacy of subsequent interventions.

Different drying techniques exhibit significant variation in their effect on pathogens, with temperature, water activity, and pretreatments (if appropriate) identified as key determinants of microbial inactivation. Once dried, pathogens may persist for extended periods; however, elevated storage temperatures and relative humidity, and pre‐drying treatments with organic acids or sulfites can enhance pathogen reduction during storage. Given the potential of recontamination during transport and handling, various pathogen‐reduction technologies for dried fruits, vegetables, herbs, and spices have been evaluated. Direct comparison among studies is challenging due to the lack of harmonized methodologies, including inoculation and recovery or detection methods. In addition to thermal treatments, other effective technologies have been investigated; however, some are restricted due to regulatory limitations related to toxic residues or other safety concerns (especially chemical treatments), whereas others face limited consumer acceptance (e.g., irradiation). Considering these constraints, no single intervention can ensure microbiological safety for these commodities while meeting regulatory requirements and maintaining the sensory and quality attributes across all dried fruits, vegetables, herbs, and spice products. Although this review and framework focused primarily on bacterial and viral hazards, fungal growth and mycotoxin production remain important considerations for certain dried and semi‐dried ingredients, particularly when water activity and storage conditions are not adequately controlled. Accordingly, the framework should be applied in conjunction with existing programs for mycotoxin prevention, monitoring, and regulatory compliance where relevant.

Considering the information reviewed and providing the reader with a structured and science‐based approach, a risk‐based framework was developed to support the evaluation and management of microbiological hazards associated with dried fruits, vegetables, herbs, and spices. The framework integrates information across the ingredient supply chain, from primary production agriculture through packaging of finished products, incorporating available data on outbreaks, prevalence, and pathogen survival. The framework is designed to assist in hazard analysis, risk assessment, and risk communication, particularly when data may be limited or uncertainty is high. At each level of the framework, pre‐defined values are assigned depending on agricultural practices, (re‐)contamination potential at different stages of production, and applied pathogen reduction steps and their controls. By application of those values, the framework can be used to determine a numerical risk score that correlates to recommended actions. When information gaps exist, conservative assumptions can be applied to support additional safety margins and protective decision‐making. The framework also highlights the need to fill important data gaps, including comprehensive prevalence and enumeration studies, improved pathogen‐detection methods for low water activity foods, and standardized approaches for evaluating the efficacy of intervention technologies.

## Author Contributions

**Heidy M. W. den Besten**: conceptualization, writing – original draft, investigation, visualization. **Linda J. Harris**: conceptualization, investigation, writing – original draft, visualization. **Jennifer C. Acuff**: conceptualization, investigation, writing – original draft, visualization. **Francois Bourdichon**: conceptualization, investigation, writing – original draft, visualization. **Rob Limburn**: conceptualization, investigation, writing – original draft, visualization. **Andreja Rajkovic**: conceptualization, investigation, writing – original draft, visualization. **Anett Winkler**: conceptualization, investigation, writing – original draft, visualization. **Polly D. Courtney**: conceptualization, investigation, writing – original draft, visualization.

## Conflicts of Interest

The authors declare no conflicts of interest.

## Supporting information




**Table S1** Interactive tool for applying the risk assessment framework for microbiological safety of dried fruits, vegetables, herbs and spices.
